# Genetic variants in microRNAs predict non-small cell lung cancer prognosis in Chinese female population in a prospective cohort study

**DOI:** 10.18632/oncotarget.13072

**Published:** 2016-11-04

**Authors:** Xia Lingzi, Yin Zhihua, Li Xuelian, Ren Yangwu, Zhang Haibo, Zhao Yuxia, Zhou Baosen

**Affiliations:** ^1^ Department of Epidemiology, China Medical University, Shenyang, Liaoning, 110122, Peoples R China; ^2^ Key Laboratory of Cancer Etiology and Prevention, China Medical University, Liaoning Province Department of Education, Shenyang, Liaoning, 110122, Peoples R China; ^3^ Department of Radiotherapy, Shenyang North Hospital, Shenyang, Liaoning, 110001, Peoples R China; ^4^ Department of Radiotherapy Oncology, The Fourth Affiliated Hospital of China Medical University, Shenyang, Liaoning, 110001, Peoples R China

**Keywords:** miRNA, SNP, lung cancer, prognosis, Chinese

## Abstract

To investigate the prognostic effect of microRNA single nucleotide polymorphisms (SNP) on non-small cell lung cancer (NSCLC) patients, 658 female participants from northeast China were enrolled in our prospective cohort study and followed up from 2010 to 2015. C-containing genotypes of miR-149 rs2292832 were associated with better overall survival (OS). The joint effect of miR-149 and miR-196a2 and the joint effect of miR-149 and miR-608 were also observed in our study. To verify the function of miR-149 rs2292832, A549 cell lines were stably transfected with lenti-virus containing miR-149-C vector, miR-149-T vector and empty vector. Cells containing C allele assumed a higher expression level of miR-149, a decrease in cell growth and the sensitivity to anticancer drug when compared with cells containing T allele. The role of miR-149 playing in cancer prognosis may function through DNA topoisomerases 1 (TOP1) pathway, according to the results from luciferase reporter assays. In conclusion, miR-149 C allele may be a prognostic biomarker for better NSCLC OS.

## INTRODUCTION

Lung cancer is a common malignancy worldwide, accounting for 23.6% deaths due to cancers. In China, the mortality of lung cancer is the highest for both men and women [1, 2]. The overall 5-year-survival is only 16.1% for Chinese female population as reported in a recent cohort study [3]. The poor survival rates for female lung cancer patients call for the refinements in clinical treatment.

Recently, microRNA has emerged as an influential factor in lung cancer progression [4]. microRNAs are a class of 19-25nt in length, small non-coding RNAs [5]. Aberrant expression profiles and genetic polymorphisms of some microRNAs have been shown to be associated with cancer survival [6–9].

Genetic variants presented in miRNA genes or genes being involved in processing mechanisms may inhibit miRNA expression [10]. Moreover, SNPs located in some specified sites may weaken the affinity between miRNAs and their target mRNAs [11]. In our present study, we aimed to investigate the effects of genetic polymorphisms in pre-miRNAs on the prognosis of non-small cell lung cancer.

## RESULTS

No statistical significance was observed for baseline characteristics between the lost to follow-up group and the follow-up group (Supplementary Table S1). The characteristics of the follow-up group were listed in Table 1. As shown in the table, the significant death rate was observed only in patients with different clinical stages (P<0.001). While, the significant median survival time (MST) can be observed in patients with different histological types, clinical stages and in patients with or without receipt of chemotherapy or surgery (P<0.05).

**Table 1:** The baseline characteristics of the non-small cell lung cancer patients

Characteristics	cases (584)	%	Deaths	%	P-value	MST (months)	Log-rank P-value
Age(yr)					0.579		0.856
≤60	252	43.1	209	82.9		25.54	
>60	332	56.9	281	84.6		25.86	
Tobaccon exposure							
Smoker	326	55.8	273	83.8	0.613	22.90	0.146
Non-smoker	258	44.2	220	85.2		18.53	
Histological type					0.406		<0.001
AD	317	54.3	260	82.0		22.79	
SQU	230	39.4	201	87.4		25.01	
others	37	6.3	29	78.4		34.80	
Clinical stage					<0.001		<0.001
I	132	22.6	94	71.2		41.56	
II	65	11.1	51	78.5		27.43	
III	309	52.9	275	89.0		18.86	
IV	78	13.4	70	89.7		23.67	
Chemotherapy					0.225		<0.001
no	55	9.4	43	78.2		15.15	
yes	529	90.6	447	84.5		27.67	
Surgery					0.218		0.005
no	200	34.2	173	86.5		22.48	
yes	384	65.8	317	82.6		27.40	

*AD: adenocarcinoma, SQU:squamous carcinoma, others: large cell carcinoma or mixed types, MST: median survival time.

**Figure 1:**
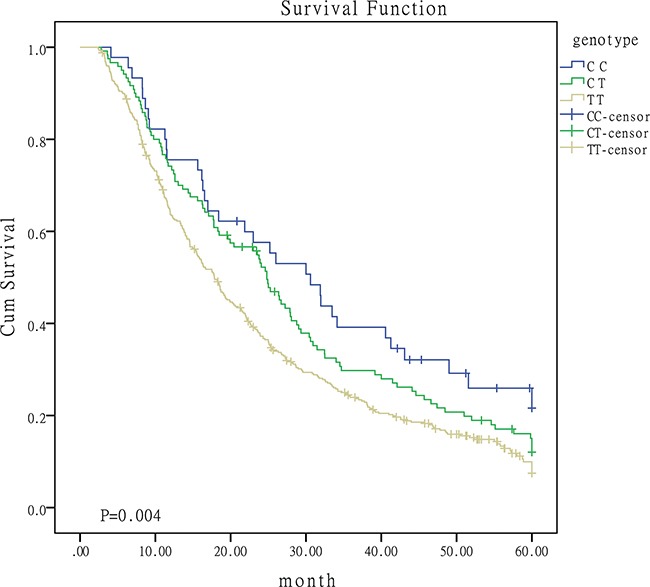
Survival curves for patients with different genotypes of miR-149

The association between the five SNPs and OS was summarized in Table 2. Patients containing C allele of miR-149 rs2292832 may have a better OS, longer MST and lower death rate. The effect of miR-149 SNPs on survival rate may be associated with the number of C alleles. Patients with more C allele may have a better prognosis. Figure 1 was the survival curves for patients with different genoytypes of miR-149 rs2292832. No statistically significant association was observed for the other four SNPs.

**Table 2:** The association between the five SNPs and OS

SNP Genotypes	cases	MST	Log-rank P-value	Crude HR	95% CI (P-value)	aHR	95%CI(P-value)
mir-149 rs2292832							
TT	419	24.08	0.004	1		1	
CT	120	28.50		0.781	0.626-0.976(0.029)	0.782	0.626-0.976(0.030)
CC	45	32.59		0.608	0.425-0.870(0.006)	0.607	0.425-0.869(0.006)
TT	419	24.08	0.002	1		1	
CC+CT	165	29.62		0.730	0.598-0.892(0.002)	0.739	0.605-0.903(0.003)
CT+TT	539	25.09	0.015	1		1	
CC	45	32.59		0.647	0.454-0.922(0.016)	0.644	0.452-0.919(0.015)
mir-146a rs2910164							
CC	189	25.68	0.983	1		1	
CG	302	25.59		0.986	0.807-1.204(0.886)	0.999	0.818-1.221(0.995)
GG	93	25.44		1.005	0.766-1.319(0.969)	0.967	0.737-1.269(0.808)
mir-196a2 rs11614913							
TT	145	24.21	0.414	1		1	
CT	319	26.07		0.916	0.738-1.136(0.424)	0.978	0.788-1.214(0.842)
CC	120	27.17		0.833	0.635-1.093(0.187)	0.854	0.651-1.121(0.255)
mir-608 rs4919510							
GG	154	24.79	0.803	1		1	
CG	308	23.53		1.070	0.836-1.370(0.590)	0.976	0.762-1.251(0.849)
CC	122	25.06		0.996	0.735-1.350(0.980)	1.015	0.749-1.375(0.925)
mir-423 rs6505162							
CC	386	24.27	0.874	1		1	
AC	181	24.22		1.010	0.805-1.269(0.929)	1.017	0.809-1.277(0.886)
AA	17	25.10		0.853	0.453-1.606(0.622)	0.953	0.506-1.796(0.881)

*MST: median survival time, aHR: HR adjusted by histological type, clinical stage, receipt of chemotherapy or surgery.

**Figure 2:**
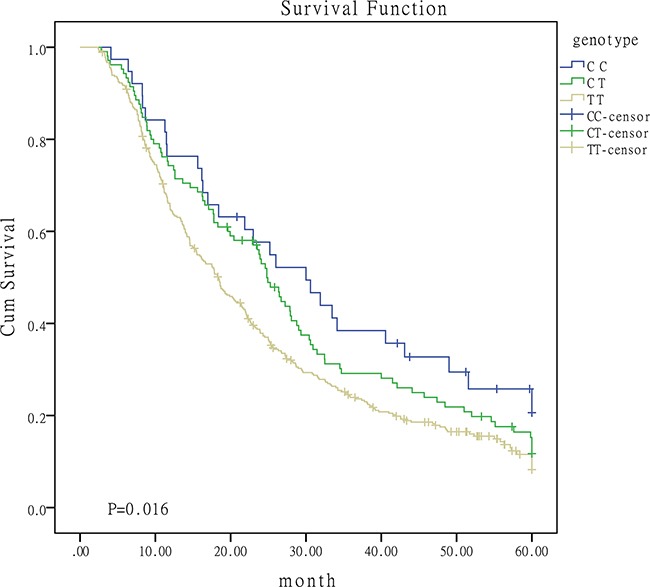
Survival curves for I/II/III patients with different genotypes of miR-149

Given the effect of patients baseline characteristics on survival, we proceeded to conduct stratified analyses. Results were summarized in Table 3. The protective role of C-containing genotypes of miR-149 were widely observed in patients from different strata. Figure 2 was the survival curves for I/II/III patients with different genotypes. The protective effect of CC genotypes in miR-196a2 rs11614913 was observed in patients with squamous carcinoma. CC genotype of miR-608 rs4919510 may function as a risk factor for patients receiving chemotherapy or surgery. No significant association was observed for miR-146a and miR-423 polymorphisms in the stratified analyses (data not shown).

**Table 3:** Stratified analyses of the association between the five SNPs and OS

SNPs subgroup	cases	Model	aHR	95%CI (P-value)
mir-149 rs2292832				
AGE>60	332	CC vs TT	0.494	0.294-0.830(0.008)
		CC+CT vs TT	0.693	0.509-0.943(0.020)
		CC vs CT+TT	0.519	0.311-0.866(0.012)
AGE≤60	252	CT vs TT	0.749	0.560-1.001(0.051)
		CC+CT vs TT	0.758	0.582-0.987(0.040)
Non-smoker	258	CC vs TT	0.376	0.205-0.691(0.002)
		CT vs TT	0.537	0.376-0.767(0.001)
Smoker	326	CC vs TT	0.619	0.392-0.976(0.039)
SQU	230	CC vs TT	0.535	0.317-0.901(0.019)
		CT vs TT	0.651	0.444-0.955(0.028)
		CC+CT vs TT	0.608	0.437-0.847(0.003)
		CC vs CT+TT	0.598	0.357-1.000(0.050)
I/II/III	509	CC vs TT	0.625	0.424-0.921(0.017)
		CT vs TT	0.795	0.625-1.011(0.062)
		CC+CT vs TT	0.746	0.600-0.926(0.008)
		CC vs CT+TT	0.662	0.451-0.972(0.035)
Chemotherapy	529	CC vs TT	0.622	0.432-0.895(0.010)
		CT vs TT	0.735	0.583-0.927(0.009)
		CC vs CT+TT	0.673	0.470-0.965(0.031)
		CC+CT vs TT	0.702	0.570-0.864(0.001)
Surgery	384	CT vs TT	0.677	0.509-0.901(0.008)
		CC vs TT	0.540	0.348-0.839(0.006)
		CC+CT vs TT	0.635	0.492-0.819(<0.001)
		CC vs CT+TT	0.598	0.387-0.923(0.020)
mir-196a2 rs11614913				
SQU	230	CC vs TT	0.608	0.384-0.963(0.034)
		CC vs CT+TT	0.679	0.463-0.996(0.047)
mir-608 rs4919510				
Chemotherapy	529	CC vs GG	1.277	1.036-1.535(0.028)
Surgery	384	CC vs GG	1.411	1.066-1.814(0.040)
		CC+CG vs GG	1.359	1.056-1.762(0.013)

*SQU: squamous carcinoma, aHR: HR adjusted by histological type, clinical stage and therapeutic strategy.

The joint effect for the miRNA SNPs was estimated in our study. Results were summarized in Table 4. As shown in the table, the joint effect of miR-149 SNP and miR-196a2 SNP appeared in patients with squamous cell carcinoma. The joint effect of miR-149 SNP and miR-608 SNP appeared in patients receiving chemotherapy or surgery.

**Table 4:** The joint effect of the miRNA SNPs on OS

Strata	miRNAs	Model	Cases	HR	95%CI	P
SQU	miR-149, miR-196a2					
		0*	42	1.00		
		1*	98	0.886	0.594-1.323	0.555
		2*	62	0.614	0.392-0.961	0.033
		3+4*	28	0.484	0.270-0.866	0.015
Chemotherapy	miR-149, miR-608					
		0^#^	75	1.00		
		1^#^	229	1.097	0.787-1.529	0.584
		2^#^	168	1.029	0.733-1.445	0.867
		3+4^#^	57	0.572	0.367-0.893	0.014
Surgery	miR-149, miR-608					
		0^#^	57	1.00		
		1^#^	164	1.241	0.857-1.798	0.253
		2^#^	120	1.139	0.774-1.676	0.509
		3+4^#^	43	0.587	0.356-0.968	0.037

* 0:TT of miR-149 and miR-196a2; 1:CT of miR-149 and TT of miR-196a2, or TT of miR-149 and CT of miR-196a2; 2: CT of miR-149 and miR-196a2, or CC of miR-149 and TT of miR-196a2, or TT of miR-149 and CC of miR-196a2; 3: CT of miR-149 and CC of miR-196a2, or CC of miR-149 and CT of miR-196a2; 4: CC of miR-149 and miR-196a2.

# 0:TT of miR-149 and CC of miR-608; 1: TT of miR-149 and CG of miR-608, or CT of miR-149 and CC of miR-608; 2: TT of miR-149 and GG of miR-608, or CC of miR-149 and CC of miR-608, or CT of miR-149 and CG of miR-608; 3: CT of miR-149 and GG of miR-608, or CC of miR-149 and CG of miR-608; 4: CC of miR-149 and GG of miR-608

SQU:squamous cell carcinoma.

To explore the impact of these five SNPs on expression levels in lung cancer tissues, we conducted the qRT-PCR and results were summarized in Supplementary Table S2. The G-containing genotypes of miR-146a, C-containing genotypes of miR-149 and miR-196 were observed to be associated with increased expression level of mature miRNA. The association between the three SNPs and miRNA expression was described in Supplementary Figure S1. No significant association with miRNA expression was observed for miR-423 and miR-608 SNPs.

The effect of rs2292832 on miR-149 expression was verified in our A549 cell lines. As shown in Supplementary Figure S2, vectors containing C allele has a higher expression level of miR-149 than those containing T allele. The expression level of vectors containing C allele was 1.55 times that of the vectors containing T allele (P<0.001).

**Figure 3:**
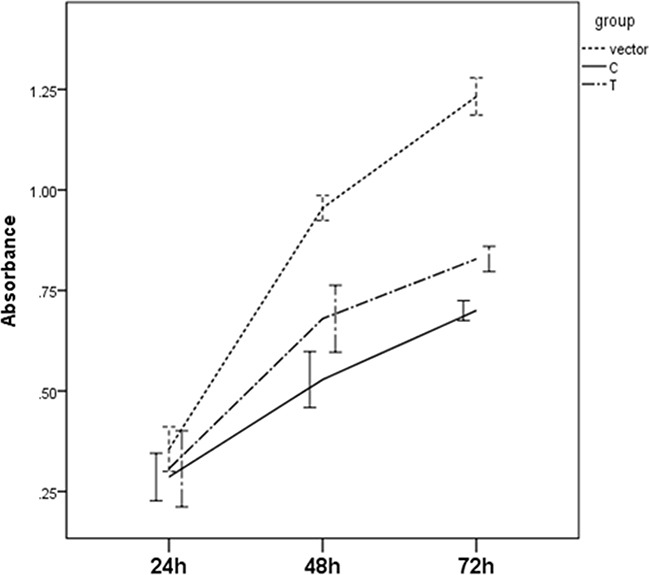
Effect of miR-149 rs2292832 on cell proliferation

**Figure 4:**
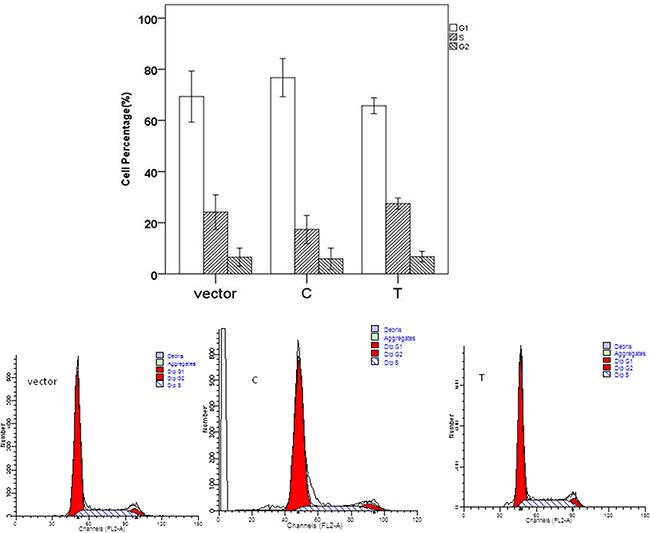
Effect of miR-149 rs2292832 on cell cycle

The significant differences for cell proliferation was observed in our study. Results were shown in Figure 3. Vectors containing C allele may lead to decreased cell proliferation when compared to vectors containing T allele (P=0.038) and empty vectors (P<0.001). These results were accordant with the results in cell cycle analyses. As shown in Figure 4, cell number of G1 phase in vectors containing C allele was higher than that in vectors containing T allele (P=0.004) and empty vectors (P=0.023). Cell number of S phase in vectors containing C allele was lower than that in vectors containing T allele (P=0.001) and empty vectors (P=0.007). These results implied that cells containing C allele may obtain inhibition at G1/S transition.

**Figure 5:**
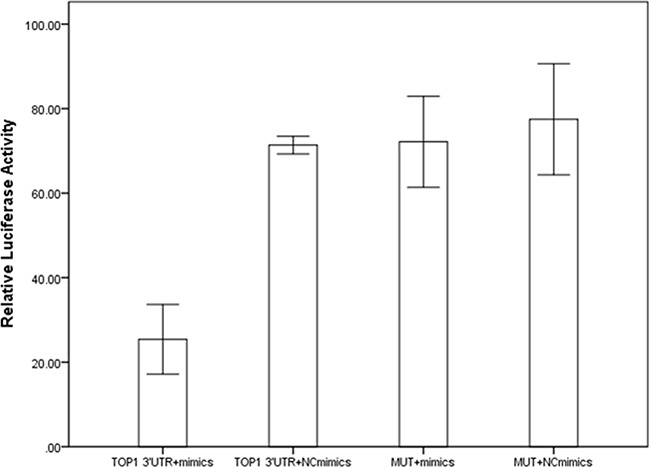
The results of luciferase reporter assay

**Figure 6:**
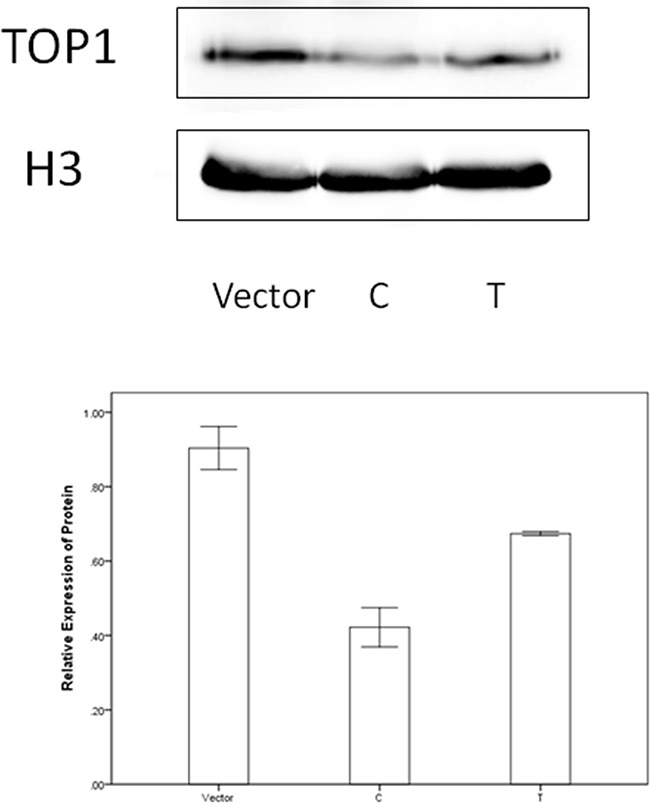
The western blotting results

DNA topoisomerases IB (TOP1) was screened out as potential target mRNA of miR-149 by bioinformatics methods. Luciferase reporter assay was constructed to investigate the influence of miR-149 in activities of target gene. Results were shown in Figure 5. The relative luciferase activities for cells transfected with TOP1-3′UTR and miR-149 mimics were lower than the other three groups (P<0.001). We proceeded to perform the qRT-PCR and western blotting to investigate the influence of miR-149 in trascription and translation of TOP1 gene. No significant effect on TOP1 mRNA levels was observed in qRT-PCR (P=0.079, Supplementary Figure S3). While, the significant effect on TOP1 protein expression was observed in western blotting (P<0.001, Figure 6). These results implied that miR-149 SNP may influence the translation of TOP1 mRNA instead of the transcription of TOP1 gene.

**Figure 7:**
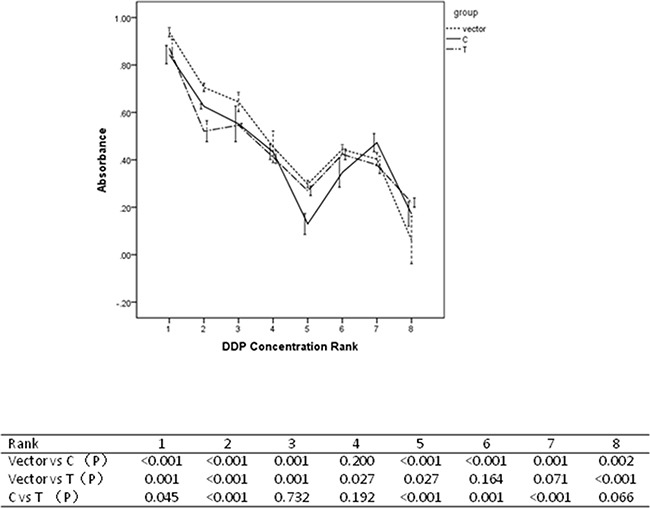
Resutls for drug sensitivity assays

miR-149 SNP conferred significant effect on survival rate for cells being treated with cis-Dichlorodiamineplatinum(II) (DDP) in our study. Results were shown in Figure 7. Being treated with concentrations of Rank 1, 5 and 6, cells containing C allele were more sensitive to DDP than those containing T allele. Being treated with concentrations of Rank 2 and 7, cells containing C allele were less sensitive to DDP than those containing T allele.

## DISCUSSION

The main results of our study is the protective effect of miR-149 SNPs on OS. Functional study results imply that C allele can increase the miR-149 expression levels, and therefore causing the decrease in TOP1 expression levels and cell growth. These results above explain the reasons that patients with C allele of miR-149 rs2292832 have better prognosis. We also observed the drug sensitivity for cells with C allele of miR-149 rs2292832 for certain DDP concentrations. These results support our observations that C allele is associated with better prognosis for patients who receiving chemotherapy.

The expression of miR-149 exhibited differently in cancer survival as previously reported [12–15]. Our results for miR-149 are consistent with Katada, Luo and Ke's studies [12–14]. Our study may help to have a deeper congnization of the role of miR-149 playing in cancer progression. Except for EMT and AKT1 signaling, miR-149 also involves in DNA topoisomerases functioning pathway to inhibit cancer cell growth. DNA topoisomerases are grouped as type I and type II. TOP1 (TOPIB) enzyme mainly involves in DNA relaxing to remove negative supercoils [16]. The inhibition of TOP1 expression may result in the increase in negative supercoils and therefore the decrease in cell growth. In our study, we observed the increase in miR-149 expression, the decrease in TOP1 protein levels and cell growth.

The prognostic role of miR-149 rs2292832 has been demonstrated previously in various types of cancer [17–24]. For NSCLC, results in our study are consistent with Hong's study [17]. For the previous study [17], the participants are early-stage, surgically resected NSCLC in Korea. While the subjects included in our study are 584 Chinese females with late-stage NSCLC. Another distinction from Hong's study is the association study for SNPs in miR-423 and miR-608 with NSCLC OS. Results in the present study support our previous study [25] and strengthen the role of miR-149 rs2292832 playing in NSCLC OS.

We also observed the significant association for miR-196a2 and miR-608 with NSCLC OS. In previous study, we noticed that the CC genotype of miR-196a2 rs11614913 is a risk factor for II/IIIA NSCLC patients [17]. While, we cannot observe the same effect in our study. In our study, we only observed the protective role of C-containing genotypes in squamous cell carcinoma. miR-608 CC genotype showed the risk effect on OS for patients receiving chemotherapy. This may indicate that miR-608 CC genotype may enhance the multi-drug resistant (MDR). While, the relatively small sample size for stratified analyses may shad the significance of the results. Larger studies are warranted to verify our results. The effect of miR-146a, miR-423 and their SNPs on cancer progression has been extensively studied previously [26–32]. Nevertheless, the effect of the two genetic variants on NSCLC OS was not observed in our study.

Some defects exist in our study. Firstly, the sample size is not large in the cohort study. And the number is even smaller for stratified analyses. Secondly, only A549 cell lines were used in functional study. Thirdly, the function for miR-149 and its SNP was verified in vitro, without being verified in vivo.

In spite of the defects above, some advantages exist in our present study. One strength is that this is a multi-center study, enhancing the reliability of the results. Another one is the use of a systematic search for the genome-wide SNPs in miRNAs. The third one is that the subjects recruited are all females. Previous studies pay more attention to men and smokers. Although women were included in their studies, the number was relatively small. Our present study aimed to help to provide evidence for detection of prognostic biomarkers for females. The fourth one is the first time to obtain the role of miR-608 SNP in NSCLC OS, to the best of our knowledge. The last but not the least, this is the first report that TOP1 is the target mRNA of miR-149, to the best of our knowledge.

In conclusion, we provided evidence that miR-149, miR-196a2 and miR-608 polymorphisms may be associated with NSCLC overall survival in Chinese females. Our results support the prognostic role of miRNAs in cancer survival and the combination of miR-149 with miR-196a2 may act as a biomarker for squamous cell lung cancer prognosis. The certain combined genotypes of miR-149 and miR-608 SNPs can help to enhance the sensitivity to anti-cancer drugs and therefore predict a better OS. Our results are to be confirmed by larger prospective studies.

## MATERIALS AND METHODS

### Study population

This study was conducted in accordance with the amended Declaration of Helsinki. The institutional review board of China Medical University approved the protocol and written informed consent was obtained from all patients. All subjects were females and they were from unrelated ethnic Han Chinese. Estimates of the exposure to environmental factors were reported in previous study [33]. Individual with a total of 100 cigarettes in her lifetime was defined as a smoker, otherwise she was considered as a non-smoker [33]. The patients were recruited during March 2010 to March 2013 at the Liaoning Cancer Hospital & Institute, Shenyang North Hospital and the fourth Affiliated Hospital of China Medical University. All patients were histologically confirmed when they were enrolled in the cohort. All subjects were interviewed and venous blood sample was obtained from each subject. Detailed background and clinical information including age, sex, tobacco exposure, TNM stage, clinical stage, histological type, receipt of chemotherapy and receipt of surgery has been collected.

Subjects received telephone follow-up every three months after being diagnosed with cancer. Data from Shenyang Center for Disease Control and Prevention (CDC) registry system were collected for death cause. Inpatient and outpatient medical records and Death Registry System of Shenyang Public Security Bureau were used to confirm the date of death. The patients were followed up to death or April 2015. Totally, we collected complete information of 584 patients. Another 74 patients were lost to follow up. In our study, the outcome event was defined as death from lung cancer. If not, the patients were considered survival no matter what the death cause was. The MST is 25.67 months in the ongoing study.

### SNP selection and genotyping

We inquired the common hsa-miRNAs in public database miRBase (version 20.0) (Supplementary Table S3). We screened out 604 SNP sites with a MAF no less than 5% in Chinese Han population according to the data available in dbSNP. For these SNPs, we performed an extensive research in HapMap and Patrocles. 13 SNPs that located in pre-miRNA or mature miRNA with a MAF>0.05 were screened out. Given the consideration of false-positive rate by using a single bioinformatics tool, we return to miRBase and dbSNP to verify the sequences of the 13 SNPs. SNPs without being verified were excluded. We got 9 sites after above steps. Furthermore, the Gibbs binding free energy (ΔG,Kcal/mol) for each pair of common and variant alleles was computed using viennaRNA software [34]. The differences of the free energies between the two alleles were calculated as ΔΔG. We ranked the values of ΔΔG, and chose the 2 Kcal/mol as cut-off. 3 SNPs had a ΔΔG≥2 Kcal/mol. Among the SNPs with a ΔΔG<2 Kcal/mol, we found two SNPs located in two pivotal sites for miRNA maturation [10, 35]. rs6505162 located within 3nt of 3′ pre-miRNA and rs4919510 located in dsRNA of pre-miRNA. Finally, we selected 5 SNPs (hsa-miR-196a2 rs11614913, hsa-miR-146a rs2910164, hsa-miR-149 rs2292832, hsa-miR-423 rs6505162 and hsa-miR-608 rs4919510) (Supplementary Table S4). The work flow of SNP selection was exhibited in Supplementary Figure S4.

Phenol-chloroform method was utilized to extract genomic DNA from peripheral blood samples. The TaqMan allelic discrimination method was used to genotype the five selected SNPs according to the protocol. The samples were read and analyzed from the ABI 7500 Fast Sequence Detection System (Applied Biosystems, Lifetechnologies, USA). The average genotype call rate for all SNPs were 99.2%. About 10% of the samples were randomly selected for confirmation by repeat genotyping, and the results were 100% concordant.

### Quantitative real time RT-PCR

Sixty lung cancer tissues were obtained from surgically removed specimens of individual cases in this study (Supplementary Table S5). Total RNAs were extracted by Trizol reagent (Thermo, USA). TaqMan MicroRNA Reverse Transcription Kit (Applied Biosystem, USA) was utilized for cDNA synthesis. TaqMan MicroRNA Assays (Applied Biosystem, USA) was utilized to quantify mature miRNA expression levels according to the protocol. U6 was used as internal references. The PCR was repeated three times for every sample. Comparative CT method was used to evaluate the miRNA expression level. 2^−ΔΔCT^ is the relative miRNA expression.

### Cell lines and culture

A549 cell lines were purchased from Shanghai Institute of Biochemistry and Cell Biology, Chinese Academy of Sciences (Shanghai, China). They were maintained in RPMI-1640 medium (Gibco, USA) with 10% fetal bovine serum (Gibco, USA), 100 U/ml penicillin sodium and 100 mg/ml streptomycin sulfate. Cell lines were cultured at 37°C with an air atmosphere containing 5% carbon dioxide. In our experiment, cells were used and passaged when they reach the logarithmic phase of growth.

### Plasmid construction, lenti-virus production and stable transfection

The common miR-149 expression construct (miR-149-T) and the variant miR-149 expression construct (miR-149-C) were generated using GV369 vector (GeneChem, China). The sequence of both constructs were confirmed by direct sequencing. An empty GV369 vector was used as negative control. All the vectors were packaged with lentivirus and stable transfection were performed according to the manufacture's precedure. The transfection efficiency was assessed by counting green fluorescent protein-positive cells four days after the transfection. 2ug/ml puromycin (Amresco, USA) were used to screen out the A549 cells transfected with miR-149-T, miR-149-C and empty vectors.

### Cell proliferation and cell cycle

To investigate the cell proliferation, cells were harvested 24h, 48h and 72h after transfection. The survival rate were determined by using the Cell Counting Kit 8 (KeyGEN BioTECH, China). The absorbance value of each well was obtained at 450nm in the Bio-Rad M680 spectrophotometer (USA). Each time point and each experiment were repeated three times.

To investigate the cell cycle, cells were harvested 48h after transfection and treated by using the Cell Cycle Detection Kit (KeyGEN BioTECH, China). DNA content was detected by flow cytometry on a FACS Calibur system (BD, USA). Results were exhibited as the percentage of cells in G1, S and G2 phases.

### Drug sensitivity assay

To investigate the drug sensitivity, cells were harvested 24h after being treated with cis-Dichlorodiamineplatinum(II) (DDP) (Sigma, USA). According to the drug concentrations, cells were grouped as eight Ranks. For Rank 1, the drug concentration was 0.977ug/ml. For Rank 2, the drug concentration was 3.906ug/ml. For Rank 3, the drug concentration was 7.813ug/ml. For Rank 4, the drug concentration was 15.625ug/ml. For Rank 5, the drug concentration was 62.5ug/ml. For Rank 6, the drug concentration was 125ug/ml. For Rank 7, the drug concentration was 250ug/ml. For Rank 8, the drug concentration was 500ug/ml. Cells treated with complete medium were used as negative controls.

### Identification of target mRNA of miR-149

Starbase v2.0 was utilized to predict the potential target mRNAs of miR-149. Starbase includes the results from five informatics databases, TargetScan, PicTar, RNA22, PITA and miRanda/mirSVR (data shown in Supplementary Table S6). The potential mRNAs with at least two databases that indicating the target site were screened out. We proceeded to investigate the mature mRNA expression levels between lung cancer and normal tissues. The mRNAs with a fold change no less than 1.3 were screened out (Supplementary Table S7). Finally, we performed GO and KEGG analyses in String database for the mRNA of interest (Supplementary Table S8).

### RNA isolation from cell lines and qRT-PCR for detection of miR-149 and TOP1 mRNA

Total RNAs were obtained with RNAiso Plus (KeyGEN BioTECH, China) following the manufacture's procedure. To detect the TOP1 mRNA expression levels, a PrimeScript RT reagent Kit with gDNA Eraser (Takara, Japan) was used for cDNA synthesis. SYBR Premix Ex Taq (Takara, Japan) was used for quantitative real time PCR and GAPDH was used as an internal control. The primers (Dingguo, China) for PCR were: TOP1-F: ACAGATGAGCAAGGAAGAG, TOP1-R: TGGAGGAGGAGAAGGAAC, Human GAPDH-F258bp: AGAAGGCTGGGGCTCATTTG, Human GAPDH-R258bp:AGGGGCCATCCACAGTCTTC. To detect the miR-149 expression levels, TaqMan MicroRNA Reverse Transcription Kit (Applied Biosystems, USA) were used for cDNA synthesis and and TaqMan MicroRNA Assays (Applied Biosystems, USA) were used for quantitative PCR. U6 was used as an internal control. The PCR was repeated three times for every sample. The relative expression was calculated by the 2^−ΔΔCt^ method.

### Western blotting

Total proteins were isolated from nucleus by Nuclear and Cytoplasmic Protein Extraction Kit (Beyotime, China) and quantified using BCA method. Protein sample was analyzed on sodium dodecyl sulfate polyacrylamide gel electrophoresis (10% seperation gel and 5% spacer gel). The antibodies were anti-TOP1, anti-H3 and anti-rabbit IgG (ProteinTech, China). The dilution ratio for anti-TOP1 was 1:2000. The dilution ratio for anti-H3 was 1:500. The dilution ratio for anti-rabbit IgG was 1:2000. The stripes were visualized by ECL methods (SuperSignal West Pico, Thermo, USA) and detected using Azure C300 biosystems (USA). The grey level values were read by Image J.

### Luciferase reporter assay

TOP1 3′UTR fragment was inserted at the XbaI site, downstream of the luciferase gene in GV272 vector (GenChem, China) (Supplementary Figure S5). A vector containing the mutant of TOP1 3′UTR fragment was constructed as negative control. The vectors expressing ranilla luciferase were constructed as inner control. All of the sequences were confirmed by direct sequencing. Plasmids were isolated from E.coli by using TaKaRa MiniBEST Plasmid Purification Kit (Takara, Japan). The miR-149 mimics and the control miRNAs duplex (named as NC mimics) were purchased from GenePharma (China). The sequences were listed in Supplementary Table S9. Transfection was performed using Lipofectamine 3000 Kit (Invitrogen) according to the manufacturer's procedure. Fourty-eight hours after the transfection, luciferase activities were measured using a Dual-Luciferase Reporter Assay System (Promega) in Synergy H1 systems (Bio Tek, USA). The relative luciferase activity was calculated as the ratio of firefly fluciferase value and ranilla luciferase value. The experiment was repeated three times.

### Statistical analysis

Goodness-of-fit chi-square test was used to compare the death rates between groups with different baseline characteristics. The OS was calculated from the date at diagnosis to the date of last follow-up or death. Differences of MST between groups were estimated by log-rank test. Survival curve was estimated by Kaplan-Meier method and analyzed by the means of log-rank test. Univariate and multivariate Cox proportional hazards regression models were used to estimate the crude and adjusted hazards ratio (HRs) and their confidence interval (CIs). Student t-test and ANOVA were used to compare the differences for continuous variables. Statistically significant P-value is less than 0.05. All of the statistical analyses were performed in SPSS 17.0 and all P-values were two-sided.

## SUPPLEMENTARY FIGURES AND TABLES










