# Supplementary material for: Genetic variants in microRNAs predict non-small cell lung cancer prognosis in Chinese female population in a prospective cohort study
Source: Oncotarget. 2016 Nov 4;7(50):83101–14. doi: 10.18632/oncotarget.13072 (PMC5347756; doi:10.18632/oncotarget.13072)
Supplement: Supplementary file 1 [file oncotarget-07-83101-s001.pdf]

Genetic variants in microRNAs predict non-small cell lung cancer prognosis in Chinese female population in a prospective cohort study

SUPPLEMENTARY FIGURES AND TABLES

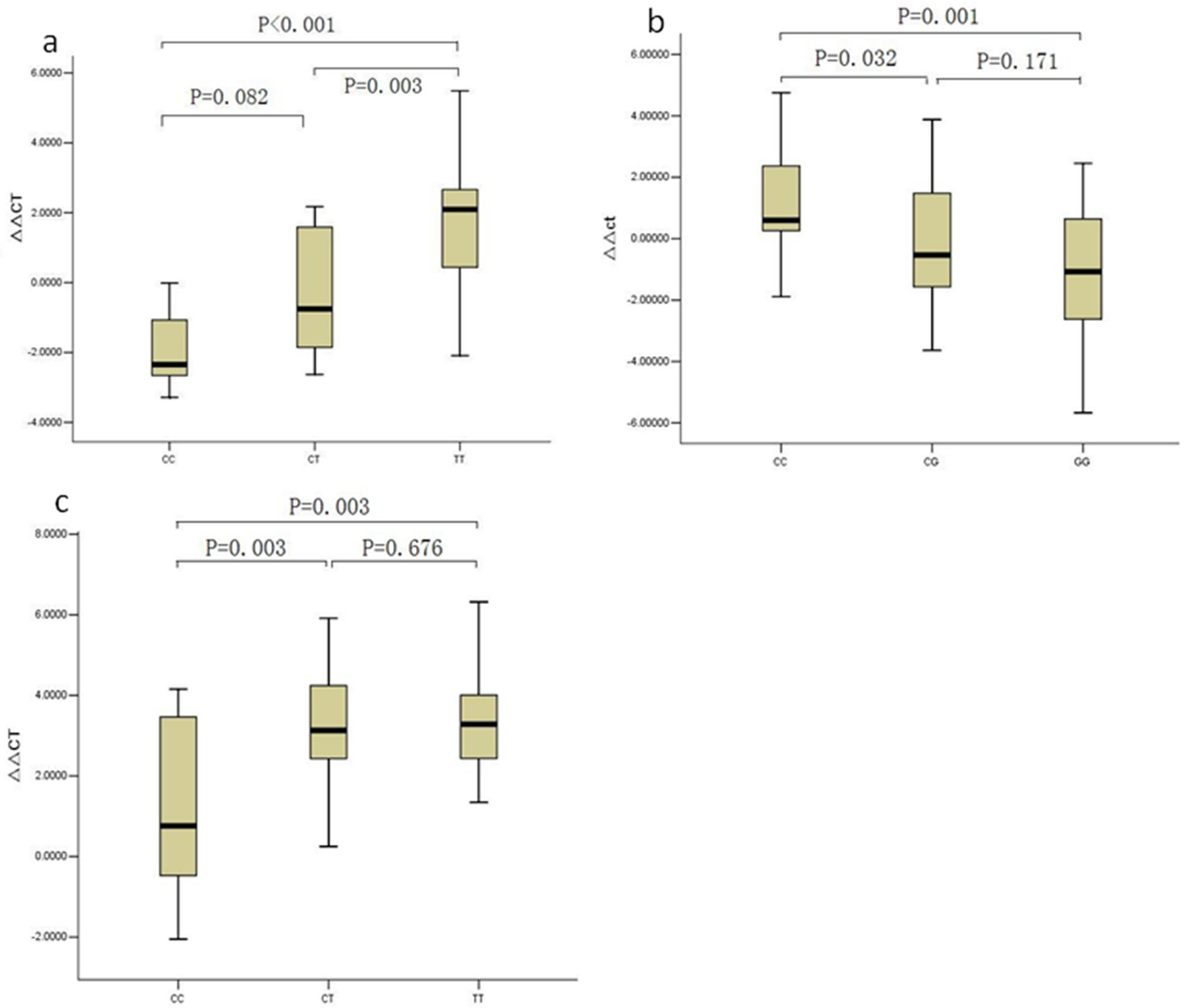

Supplementary Figure S1: The association between the three SNPs and mature miRNA expression. A. the association between rs2292832 and miR-149 expression, **B.** the association between rs2910164 and miR-146a expression, **C.** the association between rs11614913 and miR-196

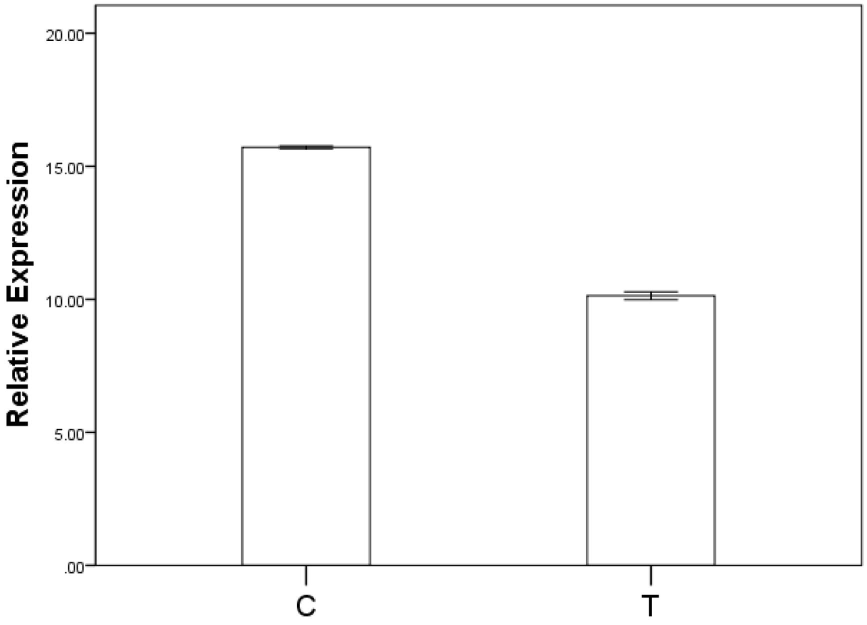

Supplementary Figure S2: The effect of rs2292832 on mature miR-149 expression.

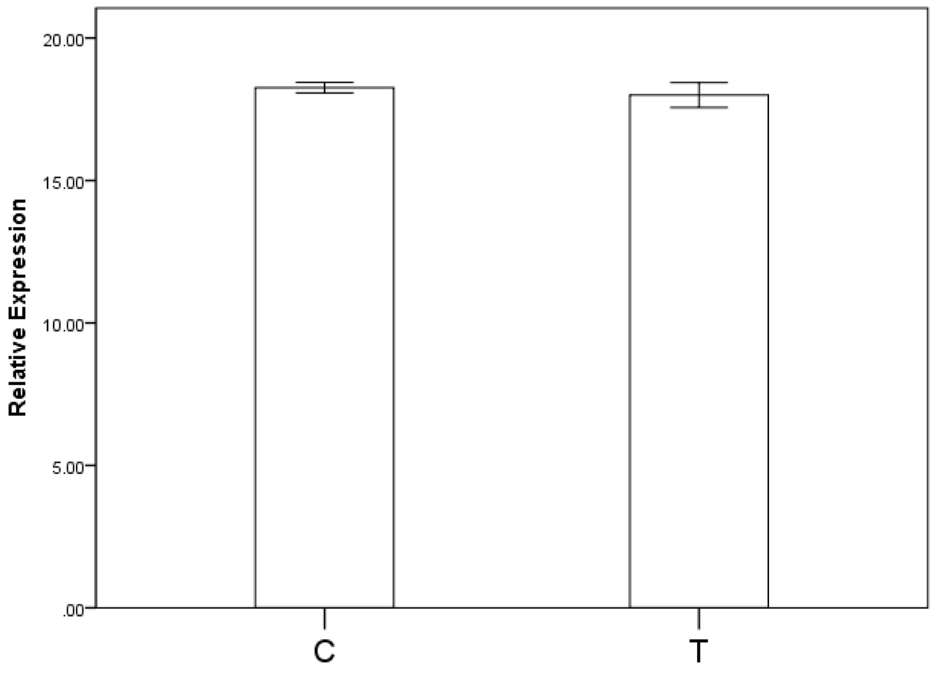

Supplementary Figure S3: The effect of miR-149 rs2292832 on TOP1 mRNA expression.

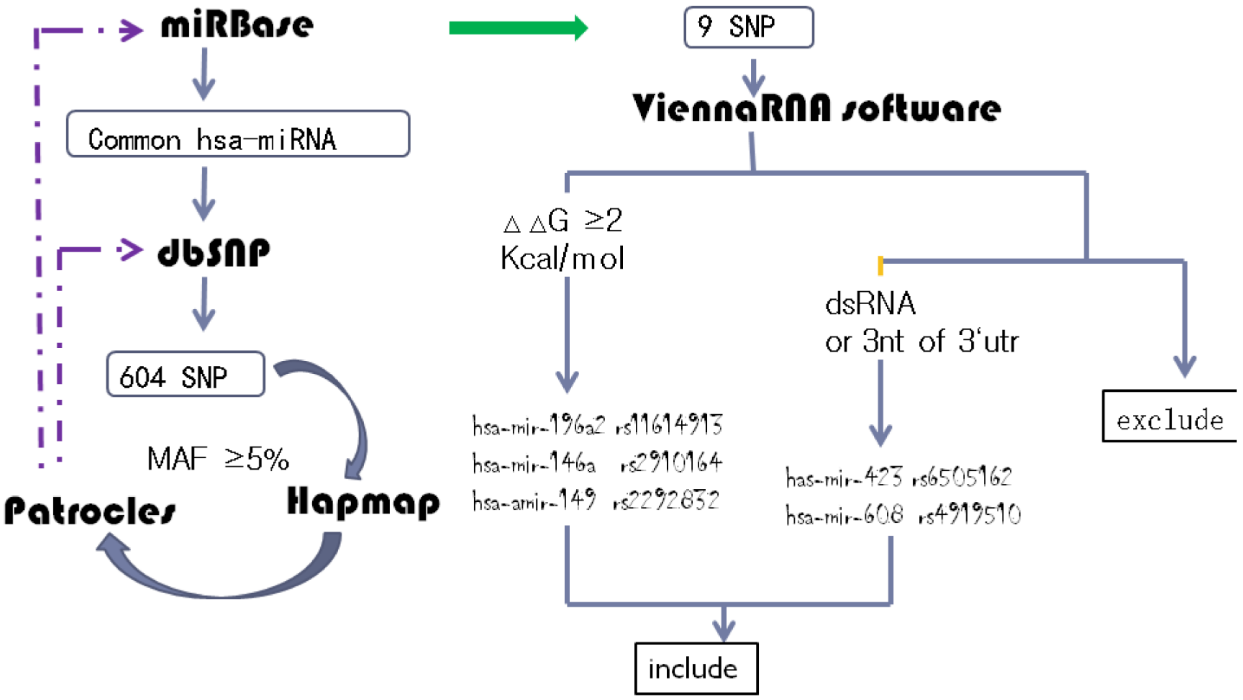

Supplementary Figure S4: The work flow of SNP selection.

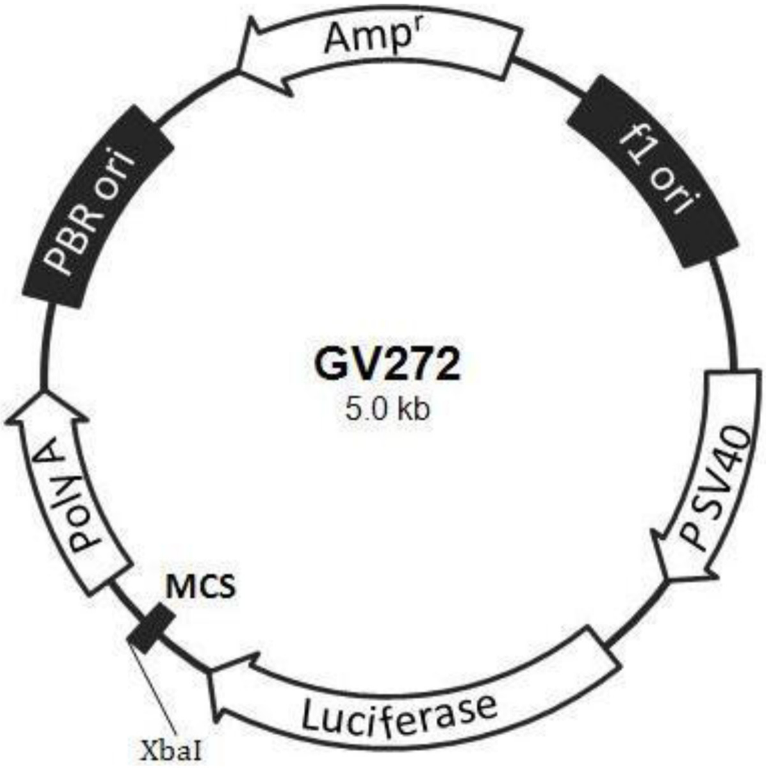

Supplementary Figure S5: The vector used for luciferase reporter assay.

Supplementary Table S1: The baseline characteristics between the lost to follow-up group and the follow-up group

variables	Lost to follow-up group	Follow-up group	p
Age ($\bar{X}$±SD)	59.45±11.41	57.12±11.47	0.101
Histological type			
AD	42	317	0.244
SQU	24	230	
others	8	37	
Clinical Stage			
I	19	132	0.124
II	11	65	
III	41	309	
IV	3	78	

Supplementary Table S2: The association of five SNPs with expression levels of mature miRNAs

miRNA	Genotype (no.)	ΔΔCT	P
miR-149	TT(40)	1.86±2.03	
	CT(13)	-0.21±1.88	0.003
	CC(7)	-1.95±1.19	<0.001
miR-146a	CC(13)	1.29±1.25	
	CG(29)	-0.15±2.09	0.032
	GG(18)	-1.14±2.52	0.001
miR-196	TT(15)	3.56±1.64	
	CT(27)	3.25±1.47	
	CC(17)	0.97±2.19	0.003
miR-423	CC(30)	0.00±2.74	
	AC(17)	1.49±1.14	0.392
	AA(13)	1.76±0.21	
miR-608	GG(11)	0.01±0.60	
	CG(38)	-1.31±1.87	0.429
	CC(11)	0.29±1.32	

Supplementary Table S3: Primary information of miRs from miRBase database

See Supplementary File 1

Supplementary Table S4: Major information of the five screened-out SNPs through bioinformation methods

miRNAs	SNPs	Bases	MAF	Location	Sequence containing SNP	△G	△△G	site
miR-196a2	rs11614913	T>C	0.341	mature	CGGCAACAAGAA ACUG[C/T]CUGAG	-51/-45.4	5.6	
miR-146a	rs2910164	C>G	0.354	mature	CCU[C/G]UGAAAUUC AGUUCUUCAG	-38.8/-41.8	3	
miR-149	rs2292832	T>C	0.354	premature	GCUGUGCUGGG GCAGC[U/C]GGA-3'	-56.1/-58.3	2.2	
miR-608	rs4919510	G>C	0.39	mature	AGGGGUGGUGUU GGGACAGCU[C/G]CGU	-33.10/-32.00	1.1	dsRNA
miR-423	rs6505162	C>A	0.122	premature	GCCCCUCAGUCUU GCUUCCUA[A/C]CC-3'	-48.6/-48.6	0	3nt of 3'UTR

MAF: minor allele frequency

Supplementary Table S5: The baseline information of the subjects included in qRT-PCR

Variants	Cases(N=60)	%
AGE	55.9±1.41	
Pathological type		
AD	24	40
SQU	22	37
Others	14	23
Stages		
I-III	37	62
IV	23	38

Supplemetary Table S6: The potential targets of miR-149 in Starbase

See Supplementary File 2

Supplementary Table S7: The fold change no less than 1.3 for mRNAs between lung cancer and normal tissues

See Supplementary File 3

Supplementary Table S8: GO and KEGG analyses for TOP1

See Supplementary File 4

Supplementary Table S9: The sequecnes of miR-149 mimics and NC mimics

Oligonucleotide	Sequence
hsa-miR-149-5p mimics 5' to 3'	UCUGGCUCCGUGUCUUCACUCCC<br>GAGUGAAGACACGGAGCCAGAUU
NC mimics 5' to 3'	Sense 5'-UUCUCCGAACGUGUCACGUTT-3<br>Antisense 5'-ACGUGACACGUUCGGAGAATT-3'
