# Supplementary material for: Genetic variants in microRNAs predict non-small cell lung cancer prognosis in Chinese female population in a prospective cohort study
Source: Oncotarget. 2016 Nov 4;7(50):83101–14. doi: 10.18632/oncotarget.13072 (PMC5347756; doi:10.18632/oncotarget.13072)
Supplement: Supplementary file 2 [file oncotarget-07-83101-s002.docx]

| **Supplementary Table 3**.Primary information of miRs from miRBase database | | | | | |
| --- | --- | --- | --- | --- | --- |
|  |  |  |  |  |  |
| **ID** | **Name** | **CHr** | **SNPs*/ID(Variation)** | | |
|  |  |  |  |  |  |
| MI0000060 | hsa-let-7a-1 | 9 | None |  |  |
| MI0000061 | hsa-let-7a-2 | 11 | None |  |  |
| MI0000062 | hsa-let-7a-3 | 22 | None |  |  |
| MI0000063 | hsa-let-7b | 22 | None |  |  |
| MI0000064 | hsa-let-7c | 21 | None |  |  |
| MI0000065 | hsa-let-7d | 9 | rs10993082(A/G) |  |  |
| MI0000066 | hsa-let-7e | 19 | rs8111742(A/G) |  |  |
| MI0000067 | hsa-let-7f-1 | 9 | None |  |  |
| MI0000068 | hsa-let-7f-2 | X | None |  |  |
| MI0000069 | hsa-mir-15a | 13 | None |  |  |
| MI0000070 | hsa-mir-16-1 | 13 | None |  |  |
| MI0000071 | hsa-mir-17 | 13 | rs4284505(A/G) |  |  |
| MI0000072 | hsa-mir-18a | 13 | rs4284505(A/G) |  |  |
| MI0000073 | hsa-mir-19a | 13 | rs4284505(A/G) |  |  |
| MI0000074 | hsa-mir-19b-1 | 13 | None |  |  |
| MI0000075 | hsa-mir-19b-2 | X | None |  |  |
| MI0000076 | hsa-mir-20a | 13 | rs4284505(A/G) |  |  |
| MI0000077 | hsa-mir-21 | 17 | rs13137(A/T) | rs1292037(A/G) |  |
| MI0000078 | hsa-mir-22 | 17 | rs11078597(C/T) | rs6502892(C/T) | rs4790812(A/G) |
| MI0000079 | hsa-mir-23a | 19 | None |  |  |
| MI0000080 | hsa-mir-24-1 | 9 | None |  |  |
| MI0000081 | hsa-mir-24-2 | 19 | None |  |  |
| MI0000082 | hsa-mir-25 | 7 | rs2261360(A/C) | rs1527423(C/T) |  |
| MI0000083 | hsa-mir-26a-1 | 3 | None |  |  |
| MI0000084 | hsa-mir-26b | 2 | rs2252235(A/G) | rs2227251(A/C) | rs3795985(C/T) |
|  |  |  | rs2227258(A/G) | rs2227255(C/T) |  |
| MI0000085 | hsa-mir-27a | 19 | None |  |  |
| MI0000086 | hsa-mir-28 | 3 | rs9859863(A/G) | CA/GC |  |
| MI0000087 | hsa-mir-29a | 7 | rs24168(C/T) | rs157907(A/G) | rs7781163(C/T) |
|  |  |  | rs157906(A/G) |  |  |
| MI0000088 | hsa-mir-30a | 6 | None |  |  |
| MI0000089 | hsa-mir-31 | 9 | rs13283671(C/T) |  |  |
| MI0000090 | hsa-mir-32 | 9 | rs10512391(A/G) | rs513852(C/G) | rs11792861(A/C) |
| MI0000091 | hsa-mir-33a | 22 | rs9620000(C/T) | rs2269661(C/G) |  |
| MI0000093 | hsa-mir-92a-1 | 13 | None |  |  |
| MI0000094 | hsa-mir-92a-2 | X | None |  |  |
| MI0000095 | hsa-mir-93 | 7 | rs2261360(A/C) | rs1527423(C/T) | rs12267(C/T) |
| MI0000097 | hsa-mir-95 | 4 | rs4696664(A/G) |  |  |
| MI0000098 | hsa-mir-96 | 7 | rs12538588(A/G) | rs4335057(A/G) |  |
| MI0000100 | hsa-mir-98 | X | rs17276588(A/G) |  |  |
| MI0000101 | hsa-mir-99a | 21 | None |  |  |
| MI0000102 | hsa-mir-100 | 11 | None |  |  |
| MI0000103 | hsa-mir-101-1 | 1 | None |  |  |
| MI0000105 | hsa-mir-29b-1 | 7 | None |  |  |
| MI0000107 | hsa-mir-29b-2 | 1 | None |  |  |
| MI0000108 | hsa-mir-103-2 | 20 | None |  |  |
| MI0000109 | hsa-mir-103-1 | 5 | None |  |  |
| MI0000111 | hsa-mir-105-1 | X | None |  |  |
| MI0000112 | hsa-mir-105-2 | X | None |  |  |
| MI0000113 | hsa-mir-106a | X | None |  |  |
| MI0000114 | hsa-mir-107 | 10 | None |  |  |
| MI0000115 | hsa-mir-16-2 | 3 | None |  |  |
| MI0000234 | hsa-mir-192 | 11 | None |  |  |
| MI0000238 | hsa-mir-196a-1 | 17 | None |  |  |
| MI0000239 | hsa-mir-197 | 1 | None |  |  |
| MI0000240 | hsa-mir-198 | 3 | None |  |  |
| MI0000242 | hsa-mir-199a-1 | 19 | None |  |  |
| MI0000251 | hsa-mir-208 | 14 | None |  |  |
| MI0000252 | hsa-mir-129-1 | 7 | None |  |  |
| MI0000253 | hsa-mir-148a | 7 | None |  |  |
| MI0000254 | hsa-mir-30c-2 | 6 | None |  |  |
| MI0000255 | hsa-mir-30d | 8 | None |  |  |
| MI0000261 | hsa-mir-139 | 11 | None |  |  |
| MI0000262 | hsa-mir-147 | 9 | None |  |  |
| MI0000263 | hsa-mir-7-1 | 9 | rs296885(C/T) |  |  |
| MI0000264 | hsa-mir-7-2 | 15 | rs1470108(G/T) | rs1470109(C/T) |  |
| MI0000265 | hsa-mir-7-3 | 19 | rs11878617(A/C) | rs879563(C/T) | rs879564(C/T) |
| MI0000266 | hsa-mir-10a | 17 | rs9907987(C/T) | rs3809782(C/T) |  |
| MI0000267 | hsa-mir-10b | 2 | None |  |  |
| MI0000268 | hsa-mir-34a | 1 | None |  |  |
| MI0000269 | hsa-mir-181a-2 | 9 | rs10760371(G/T) | rs3739760(C/T) |  |
| MI0000270 | hsa-mir-181b-1 | 1 | None |  |  |
| MI0000271 | hsa-mir-181c | 19 | None |  |  |
| MI0000272 | hsa-mir-182 | 7 | None |  |  |
| MI0000273 | hsa-mir-183 | 7 | None |  |  |
| MI0000274 | hsa-mir-187 | 18 | None |  |  |
| MI0000279 | hsa-mir-196a-2 | 12 | rs11614913(C/T) |  |  |
| MI0000281 | hsa-mir-199a-2 | 1 | None |  |  |
| MI0000282 | hsa-mir-199b | 9 | None |  |  |
| MI0000283 | hsa-mir-203 | 14 | None |  |  |
| MI0000284 | hsa-mir-204 | 9 | None |  |  |
| MI0000285 | hsa-mir-205 | 1 | None |  |  |
| MI0000286 | hsa-mir-210 | 11 | None |  |  |
| MI0000287 | hsa-mir-211 | 15 | None |  |  |
| MI0000288 | hsa-mir-212 | 17 | None |  |  |
| MI0000289 | hsa-mir-181a-1 | 1 | None |  |  |
| MI0000290 | hsa-mir-214 | 1 | None |  |  |
| MI0000291 | hsa-mir-215 | 1 | None |  |  |
| MI0000292 | hsa-mir-216a | 2 | None |  |  |
| MI0000293 | hsa-mir-217 | 2 | None |  |  |
| MI0000294 | hsa-mir-218-1 | 4 | None |  |  |
| MI0000295 | hsa-mir-218-2 | 5 | None |  |  |
| MI0000296 | hsa-mir-219-1 | 6 | None |  |  |
| MI0000297 | hsa-mir-220 | X | None |  |  |
| MI0000298 | hsa-mir-221 | X | None |  |  |
| MI0000299 | hsa-mir-222 | X | None |  |  |
| MI0000300 | hsa-mir-223 | X | None |  |  |
| MI0000301 | hsa-mir-224 | X | None |  |  |
| MI0000342 | hsa-mir-200b | 1 | None |  |  |
| MI0000433 | hsa-let-7g | 3 | None |  |  |
| MI0000434 | hsa-let-7i | 12 | None |  |  |
| MI0000437 | hsa-mir-1-2 | 18 | rs4591246(A/G) |  |  |
| MI0000438 | hsa-mir-15b | 3 | rs10936201(A/C) |  |  |
| MI0000439 | hsa-mir-23b | 9 | rs7874388(G/T) | rs10821447(A/G) | rs4743988(A/G) |
|  |  |  | rs1011784(C/G) |  |  |
| MI0000440 | hsa-mir-27b | 9 | None |  |  |
| MI0000441 | hsa-mir-30b | 8 | None |  |  |
| MI0000442 | hsa-mir-122 | 18 | rs1135519(C/T) |  |  |
| MI0000443 | hsa-mir-124-1 | 8 | None |  |  |
| MI0000444 | hsa-mir-124-2 | 8 | None |  |  |
| MI0000445 | hsa-mir-124-3 | 20 | None |  |  |
| MI0000446 | hsa-mir-125b-1 | 11 | None |  |  |
| MI0000447 | hsa-mir-128a | 2 | None |  |  |
| MI0000448 | hsa-mir-130a | 11 | None |  |  |
| MI0000449 | hsa-mir-132 | 17 | None |  |  |
| MI0000450 | hsa-mir-133a-1 | 18 | None |  |  |
| MI0000451 | hsa-mir-133a-2 | 20 | None |  |  |
| MI0000452 | hsa-mir-135a-1 | 3 | None |  |  |
| MI0000453 | hsa-mir-135a-2 | 12 | None |  |  |
| MI0000454 | hsa-mir-137 | 1 | None |  |  |
| MI0000455 | hsa-mir-138-2 | 16 | None |  |  |
| MI0000456 | hsa-mir-140 | 16 | None |  |  |
| MI0000457 | hsa-mir-141 | 12 | None |  |  |
| MI0000458 | hsa-mir-142 | 17 | None |  |  |
| MI0000459 | hsa-mir-143 | 5 | None |  |  |
| MI0000460 | hsa-mir-144 | 17 | None |  |  |
| MI0000461 | hsa-mir-145 | 5 | None |  |  |
| MI0000462 | hsa-mir-152 | 17 | None |  |  |
| MI0000463 | hsa-mir-153-1 | 2 | None |  |  |
| MI0000464 | hsa-mir-153-2 | 7 | None |  |  |
| MI0000465 | hsa-mir-191 | 3 | None |  |  |
| MI0000466 | hsa-mir-9-1 | 1 | None |  |  |
| MI0000467 | hsa-mir-9-2 | 5 | rs1501672(C/T) |  |  |
| MI0000468 | hsa-mir-9-3 | 15 | None |  |  |
| MI0000469 | hsa-mir-125a | 19 | None |  |  |
| MI0000470 | hsa-mir-125b-2 | 21 | None |  |  |
| MI0000471 | hsa-mir-126 | 9 | None |  |  |
| MI0000472 | hsa-mir-127 | 14 | None |  |  |
| MI0000473 | hsa-mir-129-2 | 11 | None |  |  |
| MI0000474 | hsa-mir-134 | 14 | None |  |  |
| MI0000475 | hsa-mir-136 | 14 | None |  |  |
| MI0000476 | hsa-mir-138-1 | 3 | None |  |  |
| MI0000477 | hsa-mir-146a | 5 | rs2910164(C/G) |  |  |
| MI0000478 | hsa-mir-149 | 2 | rs2292832(C/T) |  |  |
| MI0000479 | hsa-mir-150 | 19 | None |  |  |
| MI0000480 | hsa-mir-154 | 14 | None |  |  |
| MI0000481 | hsa-mir-184 | 15 | None |  |  |
| MI0000482 | hsa-mir-185 | 22 | None |  |  |
| MI0000483 | hsa-mir-186 | 1 | None |  |  |
| MI0000484 | hsa-mir-188 | X | None |  |  |
| MI0000486 | hsa-mir-190 | 15 | None |  |  |
| MI0000487 | hsa-mir-193a | 17 | None |  |  |
| MI0000488 | hsa-mir-194-1 | 1 | None |  |  |
| MI0000489 | hsa-mir-195 | 17 | None |  |  |
| MI0000490 | hsa-mir-206 | 6 | None |  |  |
| MI0000542 | hsa-mir-320 | 8 | None |  |  |
| MI0000650 | hsa-mir-200c | 12 | None |  |  |
| MI0000651 | hsa-mir-1-1 | 20 | rs6062238(A/G) |  |  |
| MI0000681 | hsa-mir-155 | 21 | None |  |  |
| MI0000683 | hsa-mir-181b-2 | 9 | None |  |  |
| MI0000727 | hsa-mir-128b | 3 | None |  |  |
| MI0000732 | hsa-mir-194-2 | 11 | None |  |  |
| MI0000734 | hsa-mir-106b | 7 | None |  |  |
| MI0000735 | hsa-mir-29c | 1 | None |  |  |
| MI0000736 | hsa-mir-30c-1 | 1 | None |  |  |
| MI0000737 | hsa-mir-200a | 1 | None |  |  |
| MI0000738 | hsa-mir-302a | 4 | None |  |  |
| MI0000739 | hsa-mir-101-2 | 9 | None |  |  |
| MI0000740 | hsa-mir-219-2 | 9 | None |  |  |
| MI0000742 | hsa-mir-34b | 11 | None |  |  |
| MI0000743 | hsa-mir-34c | 11 | None |  |  |
| MI0000744 | hsa-mir-299 | 14 | None |  |  |
| MI0000745 | hsa-mir-301a | 17 | None |  |  |
| MI0000746 | hsa-mir-99b | 19 | None |  |  |
| MI0000747 | hsa-mir-296 | 20 | None |  |  |
| MI0000748 | hsa-mir-130b | 22 | None |  |  |
| MI0000749 | hsa-mir-30e | 1 | None |  |  |
| MI0000750 | hsa-mir-26a-2 | 12 | None |  |  |
| MI0000760 | hsa-mir-361 | X | None |  |  |
| MI0000762 | hsa-mir-362 | X | None |  |  |
| MI0000764 | hsa-mir-363 | X | None |  |  |
| MI0000767 | hsa-mir-365-1 | 16 | None |  |  |
| MI0000769 | hsa-mir-365-2 | 17 | None |  |  |
| MI0000772 | hsa-mir-302b | 4 | None |  |  |
| MI0000773 | hsa-mir-302c | 4 | None |  |  |
| MI0000774 | hsa-mir-302d | 4 | None |  |  |
| MI0000775 | hsa-mir-367 | 4 | rs13136737(G/T) | rs7656819(C/T) |  |
| MI0000776 | hsa-mir-376c | 14 | None |  |  |
| MI0000777 | hsa-mir-369 | 14 | rs12893725(A/C) |  |  |
| MI0000778 | hsa-mir-370 | 14 | None |  |  |
| MI0000779 | hsa-mir-371 | 19 | rs3859501(A/C) | rs12460972(A/G) |  |
| MI0000780 | hsa-mir-372 | 19 | None |  |  |
| MI0000781 | hsa-mir-373 | 19 | rs12983273(C/T) | rs10425222(A/C) |  |
| MI0000782 | hsa-mir-374a | X | None |  |  |
| MI0000783 | hsa-mir-375 | 2 | None |  |  |
| MI0000784 | hsa-mir-376a-1 | 14 | None |  |  |
| MI0000785 | hsa-mir-377 | 14 | rs11160619(A/G) | rs6575812(G/T) |  |
| MI0000786 | hsa-mir-378 | 5 | rs4705365(C/T) | rs1076064(C/T) | rs1076063(A/T) |
| MI0000787 | hsa-mir-379 | 14 | rs7143098(A/G) | rs10130838(A/G) |  |
|  |  |  | rs12435162(A/T) | rs12895125(A/C) |  |
| MI0000788 | hsa-mir-380 | 14 | rs12892719(C/T) | rs12435862(A/G) |  |
| MI0000789 | hsa-mir-381 | 14 | rs10083406(A/C) |  |  |
| MI0000790 | hsa-mir-382 | 14 | rs4143957(A/G) | rs4906032(A/G) |  |
|  |  |  | rs12886869(A/G) | rs1951032(C/T) |  |
| MI0000791 | hsa-mir-383 | 8 | None |  |  |
| MI0000802 | hsa-mir-340 | 5 | rs17079966(G/T) | rs12653944(C/T) | rs6888883(C/T) |
|  |  |  | rs11249664(C/T) | rs17079966(G/T) |  |
| MI0000803 | hsa-mir-330 | 19 | rs7252448(C/T) | rs12151009(C/T) |  |
| MI0000804 | hsa-mir-328 | 16 | None |  |  |
| MI0000805 | hsa-mir-342 | 14 | None |  |  |
| MI0000806 | hsa-mir-337 | 14 | None |  |  |
| MI0000807 | hsa-mir-323 | 14 | rs7141987(C/T) | rs12586258(C/T) | rs12435862(A/G) |
| MI0000808 | hsa-mir-326 | 11 | rs476364(C/G) |  |  |
| MI0000809 | hsa-mir-151 | 8 | None |  |  |
| MI0000810 | hsa-mir-135b | 1 | None |  |  |
| MI0000811 | hsa-mir-148b | 12 | None |  |  |
| MI0000812 | hsa-mir-331 | 12 | rs11107973(C/T) |  |  |
| MI0000813 | hsa-mir-324 | 17 | None |  |  |
| MI0000814 | hsa-mir-338 | 17 | None |  |  |
| MI0000815 | hsa-mir-339 | 7 | rs2362534(A/G) | rs1057558(C/T) |  |
| MI0000816 | hsa-mir-335 | 7 | None |  |  |
| MI0000822 | hsa-mir-133b | 6 | None |  |  |
| MI0000824 | hsa-mir-325 | X | rs958410(C/T) | rs5938805(C/T) |  |
| MI0000825 | hsa-mir-345 | 14 | None |  |  |
| MI0000826 | hsa-mir-346 | 10 | rs10887569(C/T) |  |  |
| MI0001145 | hsa-mir-384 | X | rs232960(A/T) | rs232961(A/G) |  |
| MI0001150 | hsa-mir-196b | 7 | None |  |  |
| MI0001444 | hsa-mir-422a | 15 | None |  |  |
| MI0001445 | hsa-mir-423 | 17 | rs4795529(A/G) | rs6505162(A/C) |  |
| MI0001446 | hsa-mir-424 | X | rs757309(A/G) |  |  |
| MI0001448 | hsa-mir-425 | 3 | None |  |  |
| MI0001518 | hsa-mir-18b | X | None |  |  |
| MI0001519 | hsa-mir-20b | X | None |  |  |
| MI0001637 | hsa-mir-448 | X | None |  |  |
| MI0001641 | hsa-mir-429 | 1 | None |  |  |
| MI0001648 | hsa-mir-449a | 5 | None |  |  |
| MI0001652 | hsa-mir-450a-1 | X | None |  |  |
| MI0001721 | hsa-mir-431 | 14 | rs11851174(C/T) |  |  |
| MI0001723 | hsa-mir-433 | 14 | rs11623267(C/G) |  |  |
| MI0001725 | hsa-mir-329-1 | 14 | None |  |  |
| MI0001726 | hsa-mir-329-2 | 14 | None |  |  |
| MI0001727 | hsa-mir-453 | 14 | None |  |  |
| MI0001729 | hsa-mir-451 | 17 | rs901975(C/T) |  |  |
| MI0001733 | hsa-mir-452 | X | rs2256882(A/G) | rs928953(A/T) |  |
| MI0001735 | hsa-mir-409 | 14 | rs12893725(A/C) |  |  |
| MI0002464 | hsa-mir-412 | 14 | None |  |  |
| MI0002465 | hsa-mir-410 | 14 | None |  |  |
| MI0002466 | hsa-mir-376b | 14 | None |  |  |
| MI0002467 | hsa-mir-483 | 11 | rs3213225(C/T) | rs734351(C/T) |  |
|  |  |  | rs3213223(C/T) | rs3213221(C/G) |  |
| MI0002468 | hsa-mir-484 | 16 | rs12449163(C/T) |  |  |
| MI0002469 | hsa-mir-485 | 14 | rs4143957(A/G) | rs12886869(A/G) |  |
| MI0002470 | hsa-mir-486 | 8 | rs515071(C/T) | rs516946(A/G) | rs565491(C/T) |
| MI0002471 | hsa-mir-487a | 14 | None |  |  |
| MI0003123 | hsa-mir-488 | 1 | rs2223301(A/G) | rs12041859(C/T) |  |
| MI0003124 | hsa-mir-489 | 7 | rs10488542(G/T) | rs17165481(C/T) | rs1468180(A/G) |
| MI0003125 | hsa-mir-490 | 7 | rs6963819(A/G) |  |  |
| MI0003126 | hsa-mir-491 | 9 | None |  |  |
| MI0003127 | hsa-mir-511-1 | 10 | None |  |  |
| MI0003128 | hsa-mir-511-2 | 10 | None |  |  |
| MI0003129 | hsa-mir-146b | 10 | None |  |  |
| MI0003130 | hsa-mir-202 | 10 | rs2995337(A/G) | rs3008361(A/G) | rs3124440(A/T) |
|  |  |  | rs3008373(A/G) | rs4838710(G/T) |  |
| MI0003131 | hsa-mir-492 | 12 | rs2289030(C/G) | rs2289029(A/G) | rs10745704(A/G) |
|  |  |  | rs7962769(G/T) |  |  |
| MI0003132 | hsa-mir-493 | 14 | rs8021312(C/T) |  |  |
| MI0003133 | hsa-mir-432 | 14 | rs3825569(A/G) | rs6575805(A/G) |  |
| MI0003134 | hsa-mir-494 | 14 | rs9324030(A/G) | rs7158514(A/C) | rs8015713(A/G) |
| MI0003135 | hsa-mir-495 | 14 | rs8018238(C/T) | rs8004825(G/T) | rs11628379(C/T) |
|  |  |  | rs8018987(A/G) | rs2281611(A/C) |  |
| MI0003136 | hsa-mir-496 | 14 | rs4525426(A/G) | rs7342570(A/G) |  |
| MI0003137 | hsa-mir-193b | 16 | None |  |  |
| MI0003138 | hsa-mir-497 | 17 | None |  |  |
| MI0003139 | hsa-mir-181d | 19 | None |  |  |
| MI0003140 | hsa-mir-512-1 | 19 | None |  |  |
| MI0003141 | hsa-mir-512-2 | 19 | None |  |  |
| MI0003142 | hsa-mir-498 | 19 | rs7258128(C/T) |  |  |
| MI0003143 | hsa-mir-520e | 19 | None |  |  |
| MI0003144 | hsa-mir-515-1 | 19 | None |  |  |
| MI0003145 | hsa-mir-519e | 19 | None |  |  |
| MI0003146 | hsa-mir-520f | 19 | None |  |  |
| MI0003147 | hsa-mir-515-2 | 19 | None |  |  |
| MI0003148 | hsa-mir-519c | 19 | None |  |  |
| MI0003149 | hsa-mir-520a | 19 | None |  |  |
| MI0003150 | hsa-mir-526b | 19 | None |  |  |
| MI0003151 | hsa-mir-519b | 19 | None |  |  |
| MI0003152 | hsa-mir-525 | 19 | rs10417538(C/T) | rs1989486(C/T) |  |
| MI0003153 | hsa-mir-523 | 19 | None |  |  |
| MI0003154 | hsa-mir-518f | 19 | None |  |  |
| MI0003155 | hsa-mir-520b | 19 | None |  |  |
| MI0003156 | hsa-mir-518b | 19 | None |  |  |
| MI0003157 | hsa-mir-526a-1 | 19 | None |  |  |
| MI0003158 | hsa-mir-520c | 19 | None |  |  |
| MI0003159 | hsa-mir-518c | 19 | None |  |  |
| MI0003160 | hsa-mir-524 | 19 | None |  |  |
| MI0003161 | hsa-mir-517a | 19 | None |  |  |
| MI0003162 | hsa-mir-519d | 19 | None |  |  |
| MI0003163 | hsa-mir-521-2 | 19 | None |  |  |
| MI0003164 | hsa-mir-520d | 19 | None |  |  |
| MI0003165 | hsa-mir-517b | 19 | None |  |  |
| MI0003166 | hsa-mir-520g | 19 | None |  |  |
| MI0003167 | hsa-mir-516b-2 | 19 | None |  |  |
| MI0003168 | hsa-mir-526a-2 | 19 | None |  |  |
| MI0003169 | hsa-mir-518e | 19 | None |  |  |
| MI0003170 | hsa-mir-518a-1 | 19 | None |  |  |
| MI0003171 | hsa-mir-518d | 19 | None |  |  |
| MI0003172 | hsa-mir-516b-1 | 19 | None |  |  |
| MI0003173 | hsa-mir-518a-2 | 19 | None |  |  |
| MI0003174 | hsa-mir-517c | 19 | None |  |  |
| MI0003175 | hsa-mir-520h | 19 | None |  |  |
| MI0003176 | hsa-mir-521-1 | 19 | None |  |  |
| MI0003177 | hsa-mir-522 | 19 | rs305933(A/C) | rs186260(A/G) |  |
| MI0003178 | hsa-mir-519a-1 | 19 | None |  |  |
| MI0003179 | hsa-mir-527 | 19 | rs3859499(C/T) | rs3902017(C/G) | rs3902016(C/G) |
|  |  |  | rs3902015(A/G) |  |  |
| MI0003180 | hsa-mir-516a-1 | 19 | None |  |  |
| MI0003181 | hsa-mir-516a-2 | 19 | None |  |  |
| MI0003182 | hsa-mir-519a-2 | 19 | None |  |  |
| MI0003183 | hsa-mir-499 | 20 | None |  |  |
| MI0003184 | hsa-mir-500 | X | None |  |  |
| MI0003185 | hsa-mir-501 | X | None |  |  |
| MI0003186 | hsa-mir-502 | X | None |  |  |
| MI0003187 | hsa-mir-450a-2 | X | None |  |  |
| MI0003188 | hsa-mir-503 | X | rs757309(A/G) |  |  |
| MI0003189 | hsa-mir-504 | X | None |  |  |
| MI0003190 | hsa-mir-505 | X | None |  |  |
| MI0003191 | hsa-mir-513-1 | X | None |  |  |
| MI0003192 | hsa-mir-513-2 | X | None |  |  |
| MI0003193 | hsa-mir-506 | X | rs5905010(C/G) | rs5905009(C/G) | rs5905011(C/T) |
| MI0003194 | hsa-mir-507 | X | rs5905010(C/G) | rs5905009(C/G) | rs5905011(C/T) |
| MI0003195 | hsa-mir-508 | X | rs5904725(C/T) | rs5905014(C/G) |  |
| MI0003196 | hsa-mir-509-1 | X | None |  |  |
| MI0003197 | hsa-mir-510 | X | rs12008050(A/G) | rs11094416(A/C) |  |
| MI0003198 | hsa-mir-514-1 | X | None |  |  |
| MI0003199 | hsa-mir-514-2 | X | None |  |  |
| MI0003200 | hsa-mir-514-3 | X | None |  |  |
| MI0003205 | hsa-mir-532 | X | rs12014289(A/G) |  |  |
| MI0003513 | hsa-mir-455 | 9 | rs2060133(C/G) | rs2808793(A/G) |  |
| MI0003514 | hsa-mir-539 | 14 | rs12100867(C/T) | rs7161441(G/T) | rs10083406(A/C) |
| MI0003515 | hsa-mir-544 | 14 | rs12100867(C/T) | rs7161441(G/T) | rs10132916(C/T) |
|  |  |  | rs10144193(A/T) |  |  |
| MI0003516 | hsa-mir-545 | X | None |  |  |
| MI0003529 | hsa-mir-376a-2 | 14 | None |  |  |
| MI0003530 | hsa-mir-487b | 14 | None |  |  |
| MI0003556 | hsa-mir-551a | 1 | None |  |  |
| MI0003557 | hsa-mir-552 | 1 | rs4653077(A/T) | rs6664507(C/T) |  |
|  |  |  | rs2181898(C/G) | rs2147385(A/G) |  |
| MI0003558 | hsa-mir-553 | 1 | rs7514752(A/G) | rs4504908(A/G) |  |
| MI0003559 | hsa-mir-554 | 1 | rs4970956(A/G) | rs4970957(A/G) |  |
| MI0003560 | hsa-mir-92b | 1 | None |  |  |
| MI0003561 | hsa-mir-555 | 1 | None |  |  |
| MI0003562 | hsa-mir-556 | 1 | rs347283(A/G) | rs347282(A/C) | rs347285(A/G) |
|  |  |  | rs347287(C/T) | rs347286(C/G) | rs16860460(C/T) |
| MI0003563 | hsa-mir-557 | 1 | rs10753773(A/C) |  |  |
| MI0003564 | hsa-mir-558 | 2 | None |  |  |
| MI0003565 | hsa-mir-559 | 2 | None |  |  |
| MI0003566 | hsa-mir-560 | 2 | None |  |  |
| MI0003567 | hsa-mir-561 | 2 | rs7577484(C/T) | rs6434274(C/T) | rs6434275(A/G) |
|  |  |  | rs6744629(C/T) | rs13382200(C/G) |  |
|  |  |  | rs10180000(G/T) | rs6727073(C/T) |  |
| MI0003568 | hsa-mir-562 | 2 | None |  |  |
| MI0003569 | hsa-mir-563 | 3 | rs2166761(A/G) | rs1597068(A/G) | rs12485852(C/G) |
| MI0003570 | hsa-mir-564 | 3 | None |  |  |
| MI0003571 | hsa-mir-565 | 3 | None |  |  |
| MI0003572 | hsa-mir-566 | 3 | rs1005678(C/G) | rs3774752(C/T) |  |
| MI0003573 | hsa-mir-567 | 3 | rs3856723(A/G) | rs340165(C/T) |  |
| MI0003574 | hsa-mir-568 | 3 | None |  |  |
| MI0003575 | hsa-mir-551b | 3 | None |  |  |
| MI0003576 | hsa-mir-569 | 3 | rs2291900(A/G) |  |  |
| MI0003577 | hsa-mir-570 | 3 | None |  |  |
| MI0003578 | hsa-mir-571 | 4 | None |  |  |
| MI0003579 | hsa-mir-572 | 4 | rs13137396(C/T) |  |  |
| MI0003580 | hsa-mir-573 | 4 | rs6448278(C/T) |  |  |
| MI0003581 | hsa-mir-574 | 4 | None |  |  |
| MI0003582 | hsa-mir-575 | 4 | None |  |  |
| MI0003583 | hsa-mir-576 | 4 | rs6856291(A/G) |  |  |
| MI0003584 | hsa-mir-577 | 4 | rs10011293(C/T) |  |  |
| MI0003585 | hsa-mir-578 | 4 | rs12509669(A/G) | rs11100610(C/T) |  |
| MI0003586 | hsa-mir-579 | 5 | rs2910931(A/T) | rs2330982(A/T) | rs2910932(C/T) |
| MI0003587 | hsa-mir-580 | 5 | rs10071838(C/T) | rs12655052(C/T) | rs10941274(C/T) |
|  |  |  | rs3898058(A/G) | rs33670(A/C) |  |
| MI0003588 | hsa-mir-581 | 5 | rs809798(A/G) | rs788516(C/G) | rs787543(C/G) |
|  |  |  | rs788515(A/T) |  |  |
| MI0003589 | hsa-mir-582 | 5 | rs4700340(C/T) | rs17742120(A/G) |  |
| MI0003590 | hsa-mir-583 | 5 | None |  |  |
| MI0003591 | hsa-mir-584 | 5 | rs12513554(A/G) | rs7705960(C/T) | rs40522(C/T) |
| MI0003592 | hsa-mir-585 | 5 | rs11134566(A/G) |  |  |
| MI0003593 | hsa-mir-548a-1 | 6 | None |  |  |
| MI0003594 | hsa-mir-586 | 6 | rs1285014(A/G) | rs1285013(A/C) |  |
| MI0003595 | hsa-mir-587 | 6 | rs1884270(A/G) |  |  |
| MI0003596 | hsa-mir-548b | 6 | None |  |  |
| MI0003597 | hsa-mir-588 | 6 | None |  |  |
| MI0003598 | hsa-mir-548a-2 | 6 | None |  |  |
| MI0003599 | hsa-mir-589 | 7 | None |  |  |
| MI0003600 | hsa-mir-550-1 | 7 | None |  |  |
| MI0003601 | hsa-mir-550-2 | 7 | None |  |  |
| MI0003602 | hsa-mir-590 | 7 | rs2272569(A/G) |  |  |
| MI0003603 | hsa-mir-591 | 7 | rs11772017(G/T) |  |  |
| MI0003604 | hsa-mir-592 | 7 | rs951111(C/T) | rs3779538(C/T) | rs17866413(A/G) |
|  |  |  | rs11563749(A/T) | rs11563505(C/T) | rs11563750(A/C) |
|  |  |  | rs7795745(A/G) | rs6467109(A/G) | rs6467108(C/T) |
| MI0003605 | hsa-mir-593 | 7 | rs322828(A/G) | rs322825(C/T) |  |
|  |  |  | rs322827(A/C) | rs322826(C/T) |  |
| MI0003607 | hsa-mir-595 | 7 | rs4909239(A/G) | rs12670231(A/G) | rs4909238(C/T) |
|  |  |  | rs10261537(A/G) | rs10232620(C/T) |  |
| MI0003608 | hsa-mir-596 | 8 | None |  |  |
| MI0003609 | hsa-mir-597 | 8 | rs4841211(A/G) |  |  |
| MI0003610 | hsa-mir-598 | 8 | None |  |  |
| MI0003611 | hsa-mir-599 | 8 | rs16897440(C/T) |  |  |
| MI0003612 | hsa-mir-548a-3 | 8 | None |  |  |
| MI0003613 | hsa-mir-600 | 9 | None |  |  |
| MI0003614 | hsa-mir-601 | 9 | rs7865976(C/T) | rs10986008(C/T) | rs10986009(A/T) |
| MI0003615 | hsa-mir-602 | 9 | None |  |  |
| MI0003616 | hsa-mir-603 | 10 | rs1336188(C/T) | rs17505709(A/C) | rs12219473(A/G) |
|  |  |  | rs17583862(A/G) | rs10764458(C/T) |  |
| MI0003617 | hsa-mir-604 | 10 | rs7904687(A/G) | rs4749447(A/G) | rs11598940(A/G) |
|  |  |  | rs11592360(A/G) | rs4747662(A/G) |  |
| MI0003618 | hsa-mir-605 | 10 | rs10490971(A/G) | rs12251902(C/T) |  |
|  |  |  | rs2043555(A/C) | rs1915681(C/T) |  |
| MI0003619 | hsa-mir-606 | 10 | rs3001914(C/G) |  |  |
| MI0003620 | hsa-mir-607 | 10 | None |  |  |
| MI0003621 | hsa-mir-608 | 10 | rs4919510(C/G) |  |  |
| MI0003622 | hsa-mir-609 | 10 | rs11191952(C/T) | rs500144(C/T) | rs7909741(C/T) |
| MI0003623 | hsa-mir-610 | 11 | rs7944852(A/T) |  |  |
| MI0003624 | hsa-mir-611 | 11 | rs174538(A/G) |  |  |
| MI0003625 | hsa-mir-612 | 11 | rs1144925(C/T) | rs3741384(C/T) | rs11605125(C/G) |
| MI0003626 | hsa-mir-613 | 12 | rs10744003(A/T) |  |  |
| MI0003627 | hsa-mir-614 | 12 | rs1148732(C/G) |  |  |
| MI0003628 | hsa-mir-615 | 12 | rs2071449(A/C) |  |  |
| MI0003629 | hsa-mir-616 | 12 | None |  |  |
| MI0003630 | hsa-mir-548c | 12 | None |  |  |
| MI0003631 | hsa-mir-617 | 12 | rs10506837(C/T) | rs1495334(A/G) |  |
| MI0003632 | hsa-mir-618 | 12 | rs1716543(A/C) |  |  |
| MI0003633 | hsa-mir-619 | 12 | rs2052411(C/T) |  |  |
| MI0003634 | hsa-mir-620 | 12 | None |  |  |
| MI0003635 | hsa-mir-621 | 13 | rs2004000(C/T) | rs9549293(A/G) | rs9549293(A/G) |
| MI0003636 | hsa-mir-622 | 13 | rs7989199(A/G) |  |  |
| MI0003637 | hsa-mir-623 | 13 | rs731150(A/G) | rs7993715(C/G) |  |
| MI0003638 | hsa-mir-624 | 14 | rs179724(G/T) |  |  |
| MI0003639 | hsa-mir-625 | 14 | rs12434585(A/G) |  |  |
| MI0003640 | hsa-mir-626 | 15 | rs7169496(A/G) |  |  |
| MI0003641 | hsa-mir-627 | 15 | rs629255(A/G) | rs2620380(C/T) |  |
| MI0003642 | hsa-mir-628 | 15 | None |  |  |
| MI0003643 | hsa-mir-629 | 15 | rs12913478(A/G) |  |  |
| MI0003644 | hsa-mir-630 | 15 | None |  |  |
| MI0003645 | hsa-mir-631 | 15 | rs4462560(C/G) |  |  |
| MI0003646 | hsa-mir-33b | 17 | None |  |  |
| MI0003647 | hsa-mir-632 | 17 | None |  |  |
| MI0003648 | hsa-mir-633 | 17 | None |  |  |
| MI0003649 | hsa-mir-634 | 17 | rs6504458(A/G) | rs9916520(C/T) | rs9901261(C/G) |
|  |  |  | rs4144640(C/T) | rs9889353(A/G) |  |
| MI0003650 | hsa-mir-635 | 17 | rs12948224(C/G) |  |  |
| MI0003651 | hsa-mir-636 | 17 | rs3744061(A/G) |  |  |
| MI0003652 | hsa-mir-637 | 19 | None |  |  |
| MI0003653 | hsa-mir-638 | 19 | None |  |  |
| MI0003654 | hsa-mir-639 | 19 | rs7249458(A/T) |  |  |
| MI0003655 | hsa-mir-640 | 19 | rs2916076(C/T) | rs2965183(C/T) | rs4808199(A/G) |
| MI0003656 | hsa-mir-641 | 19 | rs11880261(C/T) |  |  |
| MI0003657 | hsa-mir-642 | 19 | None |  |  |
| MI0003658 | hsa-mir-643 | 19 | rs41497444(A/C) |  |  |
| MI0003659 | hsa-mir-644 | 20 | rs7269526(C/G) |  |  |
| MI0003660 | hsa-mir-645 | 20 | None |  |  |
| MI0003661 | hsa-mir-646 | 20 | rs6027483(C/G) | rs745942(C/G) | rs6027486(C/T) |
| MI0003662 | hsa-mir-647 | 20 | None |  |  |
| MI0003663 | hsa-mir-648 | 22 | None |  |  |
| MI0003664 | hsa-mir-649 | 22 | rs5752500(C/T) |  |  |
| MI0003665 | hsa-mir-650 | 22 | rs7287265(A/T) |  |  |
| MI0003666 | hsa-mir-651 | X | rs1037521(A/T) |  |  |
| MI0003667 | hsa-mir-652 | X | rs12841904(A/G) | rs5985440(A/G) | rs5985439(C/T) |
| MI0003668 | hsa-mir-548d-1 | 8 | None |  |  |
| MI0003669 | hsa-mir-661 | 8 | rs7464572(C/G) |  |  |
| MI0003670 | hsa-mir-662 | 16 | rs1135210(A/G) | rs1057147(A/G) |  |
| MI0003671 | hsa-mir-548d-2 | 17 | None |  |  |
| MI0003672 | hsa-mir-663 | 20 | None |  |  |
| MI0003673 | hsa-mir-449b | 5 | None |  |  |
| MI0003674 | hsa-mir-653 | 7 | rs7787488(C/T) | rs17165481(C/T) |  |
| MI0003675 | hsa-mir-411 | 14 | rs12892719(C/T) | rs7154535(A/G) | rs12435162(A/T) |
| MI0003676 | hsa-mir-654 | 14 | rs11621499(A/G) |  |  |
| MI0003677 | hsa-mir-655 | 14 | rs10132916(C/T) | rs10144193(A/T) |  |
| MI0003678 | hsa-mir-656 | 14 | rs8015875(A/G) | rs8016185(C/T) |  |
| MI0003679 | hsa-mir-549 | 15 | rs12372944(C/T) |  |  |
| MI0003681 | hsa-mir-657 | 17 | None |  |  |
| MI0003682 | hsa-mir-658 | 22 | rs6000905(A/G) |  |  |
| MI0003683 | hsa-mir-659 | 22 | rs5750504(A/T) |  |  |
| MI0003684 | hsa-mir-660 | X | None |  |  |
| MI0003685 | hsa-mir-421 | X | rs1341326(A/G) |  |  |
| MI0003686 | hsa-mir-542 | X | None |  |  |
| MI0003757 | hsa-mir-758 | 14 | rs7141987(C/T) | rs1885068(A/C) |  |
|  |  |  | rs12435862(A/G) | rs12586258(C/T) |  |
| MI0003760 | hsa-mir-671 | 7 | rs2487154(C/G) | rs1870238(C/G) | rs2446065(C/G)_ |
| MI0003761 | hsa-mir-668 | 14 | rs4143957(A/G) | rs12886869(A/G) |  |
| MI0003763 | hsa-mir-767 | X | rs5970293(C/G) | rs6526104(A/C) |  |
| MI0003820 | hsa-mir-454 | 17 | None |  |  |
| MI0003834 | hsa-mir-769 | 19 | rs2302788(A/G) |  |  |
| MI0003836 | hsa-mir-766 | X | rs5909648(A/C) | rs5957202(A/C) | rs6646439(A/T) |
| MI0003906 | hsa-mir-802 | 21 | rs2835104(A/G) |  |  |
| MI0005116 | hsa-mir-765 | 1 | rs945508(A/G) |  |  |
| MI0005117 | hsa-mir-768 | 16 | rs7185997(C/T) | rs11866559(A/G) |  |
| MI0005118 | hsa-mir-770 | 14 | rs2295389(C/G) | rs3783357(A/G) |  |
| MI0005202 | hsa-mir-801 | 1 | None |  |  |
| MI0005416 | hsa-mir-675 | 11 | rs2251375(A/C) | rs2067051(A/G) | rs2839698(C/T) |
| MI0005523 | hsa-mir-298 | 20 | rs13042263(C/T) | rs6026544(C/T) |  |
|  |  |  | rs6070625(C/G) | rs3746709(A/G) |  |
| MI0005524 | hsa-mir-891a | X | None |  |  |
| MI0005525 | hsa-mir-300 | 14 | None |  |  |
| MI0005527 | hsa-mir-886 | 5 | rs2346019(A/G) | rs917303(C/T) | rs4976364(A/C) |
| MI0005528 | hsa-mir-892a | X | None |  |  |
| MI0005529 | hsa-mir-220b | 19 | None |  |  |
| MI0005530 | hsa-mir-509-2 | X | None |  |  |
| MI0005531 | hsa-mir-450b | X | None |  |  |
| MI0005532 | hsa-mir-874 | 5 | rs3798138(A/G) |  |  |
| MI0005533 | hsa-mir-890 | X | rs5965660(G/T) | rs4827678(A/G) | rs4827677(C/G) |
|  |  |  | rs12015068(C/G) | rs5919984(C/T) |  |
| MI0005534 | hsa-mir-891b | X | None |  |  |
| MI0005536 | hsa-mir-220c | 19 | None |  |  |
| MI0005537 | hsa-mir-888 | X | None |  |  |
| MI0005538 | hsa-mir-892b | X | None |  |  |
| MI0005539 | hsa-mir-541 | 14 | rs4906034(A/G) | rs4906033(A/G) | rs8003408(A/G) |
|  |  |  | rs7161194(A/G) | rs12893725(A/C) | rs11160619(A/G) |
|  |  |  | rs8003403(A/G) |  |  |
| MI0005540 | hsa-mir-889 | 14 | rs12100867(C/T) | rs7161441(G/T) | rs10132916(C/T) |
|  |  |  | rs10083406(A/C) |  |  |
| MI0005541 | hsa-mir-875 | 8 | rs16897440(C/T) |  |  |
| MI0005542 | hsa-mir-876 | 9 | rs320939(G/T) | rs320938(A/G) |  |
| MI0005543 | hsa-mir-708 | 11 | rs1511237(A/G) |  |  |
| MI0005544 | hsa-mir-147b | 15 | None |  |  |
| MI0005545 | hsa-mir-190b | 1 | None |  |  |
| MI0005559 | hsa-mir-744 | 17 | rs9907196(C/T) | rs9788973(C/T) | rs8069300(C/G) |
|  |  |  | rs11654465(C/T) |  |  |
| MI0005560 | hsa-mir-885 | 3 | rs61609691(A/G) | rs9860273(C/T) |  |
|  |  |  | rs3774156(A/G) | rs3774157(A/G) |  |
| MI0005561 | hsa-mir-877 | 6 | rs1264439(G/T) | rs1264440(C/T) |  |
| MI0005562 | hsa-mir-887 | 5 | None |  |  |
| MI0005563 | hsa-mir-665 | 14 | rs1884536(C/G) |  |  |
| MI0005564 | hsa-mir-873 | 9 | rs6476104(A/G) | rs10812881(C/T) | rs2164073(A/G) |
|  |  |  | rs16913809(G/T) |  |  |
| MI0005565 | hsa-mir-543 | 14 | None |  |  |
| MI0005566 | hsa-mir-374b | X | None |  |  |
| MI0005567 | hsa-mir-760 | 1 | rs2391315(C/G) |  |  |
| MI0005568 | hsa-mir-301b | 22 | None |  |  |
| MI0005569 | hsa-mir-216b | 2 | None |  |  |
| MI0005570 | hsa-mir-208b | 14 | None |  |  |
| MI0005712 | hsa-mir-920 | 12 | None |  |  |
| MI0005713 | hsa-mir-921 | 1 | rs10800198(C/T) | rs1376660(C/T) |  |
| MI0005714 | hsa-mir-922 | 3 | None |  |  |
| MI0005716 | hsa-mir-924 | 18 | rs1945018(A/G) | rs2726263(A/G) |  |
| MI0005717 | hsa-mir-509-3 | X | None |  |  |
| MI0005755 | hsa-mir-933 | 2 | rs268214(C/T) | rs166531(C/T) |  |
| MI0005756 | hsa-mir-934 | X | None |  |  |
| MI0005757 | hsa-mir-935 | 19 | rs4806677(A/C) |  |  |
| MI0005758 | hsa-mir-936 | 10 | None |  |  |
| MI0005759 | hsa-mir-937 | 8 | None |  |  |
| MI0005760 | hsa-mir-938 | 10 | rs2505903(C/T) | rs2488677(C/T) | rs2488676(A/T) |
|  |  |  | rs2479691(C/T) |  |  |
| MI0005761 | hsa-mir-939 | 8 | None |  |  |
| MI0005762 | hsa-mir-940 | 16 | rs12386039(A/G) |  |  |
| MI0005763 | hsa-mir-941-1 | 20 | rs2427556 |  |  |
| MI0005764 | hsa-mir-941-2 | 20 | rs34604519 |  |  |
| MI0005765 | hsa-mir-941-3 | 20 | rs12625445 |  |  |
| MI0005767 | hsa-mir-942 | 1 | rs1289667(C/T) | rs2057594(A/G) | rs10494197(C/T) |
| MI0005768 | hsa-mir-943 | 4 | rs1077020 |  |  |
| MI0005769 | hsa-mir-944 | 3 | rs1515492(A/G) | rs9873617(C/G) | rs1515491(C/T) |
| MI0005775 | hsa-mir-297 | 4 | rs3866823(A/T) | rs4115271(C/T) | rs4115272(C/T) |
| MI0006271 | hsa-mir-1178 | 12 | None |  |  |
| MI0006272 | hsa-mir-1179 | 15 | rs7162568(G/T) | rs8023916(C/T) |  |
| MI0006274 | hsa-mir-1181 | 19 | rs2129944(A/C) |  |  |
| MI0006275 | hsa-mir-1182 | 1 | rs10864654(A/G) | rs4304564(C/T) |  |
|  |  |  | rs7513011(A/T) | rs4409638(A/G) |  |
| MI0006276 | hsa-mir-1183 | 7 | rs17144483(C/T) | rs3807643(C/G) |  |
| MI0006277 | hsa-mir-1184-1 | X | None |  |  |
| MI0006311 | hsa-mir-1225 | 16 | None |  |  |
| MI0006313 | hsa-mir-1226 | 3 | None |  |  |
| MI0006316 | hsa-mir-1227 | 19 | None |  |  |
| MI0006318 | hsa-mir-1228 | 12 | rs1800187(C/T) | rs1800141(A/G) |  |
| MI0006319 | hsa-mir-1229 | 5 | rs166624(G/T) |  |  |
| MI0006321 | hsa-mir-1231 | 1 | rs4379719(A/G) | rs2820289(C/T) | rs2292822(C/G) |
| MI0006323 | hsa-mir-1233-1 | 15 | None |  |  |
| MI0006324 | hsa-mir-1234 | 8 | None |  |  |
| MI0006326 | hsa-mir-1236 | 6 | rs34555781(A/C) | rs9501161(A/G) | rs403569(C/T) |
| MI0006327 | hsa-mir-1237 | 11 | rs645078(A/C) | rs508168(A/G) |  |
| MI0006328 | hsa-mir-1238 | 19 | rs8108051(C/T) |  |  |
| MI0006332 | hsa-mir-1200 | 7 | rs17418220(C/T) | rs10232938(A/G) |  |
| MI0006334 | hsa-mir-1202 | 6 | rs9371408(C/T) |  |  |
| MI0006335 | hsa-mir-1203 | 17 | rs11079811(A/G) |  |  |
| MI0006336 | hsa-mir-663b | 2 | None |  |  |
| MI0006337 | hsa-mir-1204 | 8 | None |  |  |
| MI0006338 | hsa-mir-1205 | 8 | rs9649959(A/G) | rs7010121(A/G) |  |
| MI0006339 | hsa-mir-1206 | 8 | rs4733827(A/G) | rs4733826(A/G) | rs4733825(C/T) |
|  |  |  | rs1863564(C/T) | rs1863563(A/G) | rs7017456(G/T) |
|  |  |  | rs7014626(C/G) | rs6996633(A/T) |  |
| MI0006340 | hsa-mir-1207 | 8 | rs2720658(A/T) | rs2720659(A/G) | rs2648861(A/G) |
|  |  |  | rs10106014(C/G) | rs2720660(A/G) | rs2648862(A/C) |
| MI0006341 | hsa-mir-1208 | 8 | rs10956412(A/C) | rs7814495(C/G) | rs2648842(C/T) |
| MI0006344 | hsa-mir-548e | 10 | rs1927159(A/C) | rs12248431(A/G) |  |
| MI0006345 | hsa-mir-548j | 22 | rs4820685(A/G) |  |  |
| MI0006346 | hsa-mir-1285-1 | 7 | None |  |  |
| MI0006347 | hsa-mir-1285-2 | 2 | rs2592183(A/G) |  |  |
| MI0006348 | hsa-mir-1286 | 22 | None |  |  |
| MI0006349 | hsa-mir-1287 | 10 | rs11189590(G/T) | rs7924303(C/T) | rs7072216(C/T) |
|  |  |  | rs17109634(C/T) | rs10883085(A/T) | rs7908310(A/G) |
|  |  |  | rs10883087(A/T) | rs6584193(C/T) | rs11189592(G/T) |
| MI0006350 | hsa-mir-1289-1 | 20 | None |  |  |
| MI0006351 | hsa-mir-1289-2 | 5 | rs7730256(C/T) | rs10060919(C/G) |  |
| MI0006352 | hsa-mir-1290 | 1 | rs7554974(C/T) |  |  |
| MI0006353 | hsa-mir-1291 | 12 | rs3741628(G/T) |  |  |
| MI0006354 | hsa-mir-548k | 11 | None |  |  |
| MI0006355 | hsa-mir-1293 | 12 | rs11169331(C/T) | rs11169332(A/G) | rs10783342(C/T) |
| MI0006356 | hsa-mir-1294 | 5 | None |  |  |
| MI0006357 | hsa-mir-1295a | 1 | rs10911192(A/C) |  |  |
| MI0006358 | hsa-mir-1297 | 13 | rs4884963(C/T) | rs9536676(A/G) | rs4351942(A/C) |
| MI0006359 | hsa-mir-1299 | 9 | None |  |  |
| MI0006361 | hsa-mir-548l | 11 | None |  |  |
| MI0006362 | hsa-mir-1302-1 | 12 | None |  |  |
| MI0006363 | hsa-mir-1302-2 | 1 | None |  |  |
| MI0006364 | hsa-mir-1302-3 | 2 | None |  |  |
| MI0006365 | hsa-mir-1302-4 | 2 | rs10173558(C/T) | rs7577873(A/G) |  |
| MI0006366 | hsa-mir-1302-5 | 20 | rs2426169(A/G) |  |  |
| MI0006367 | hsa-mir-1302-6 | 7 | RS1637634(A/G) | rs12533070(A/C) | rs17138628(A/G) |
| MI0006368 | hsa-mir-1302-7 | 8 | None |  |  |
| MI0006369 | hsa-mir-1302-8 | 9 | rs7041222(A/C) |  |  |
| MI0006370 | hsa-mir-1303 | 5 | rs4958761(A/G) | rs4958760(A/G) | rs4958759(C/G) |
|  |  |  | rs4958382(C/T) |  |  |
| MI0006371 | hsa-mir-1304 | 11 | None |  |  |
| MI0006372 | hsa-mir-1305 | 4 | None |  |  |
| MI0006373 | hsa-mir-1243 | 4 | rs1385662(C/T) | rs13135004(A/T) | rs13134980(C/T) |
|  |  |  | rs17625836(A/G) | rs4834315(A/G) |  |
| MI0006374 | hsa-mir-548f-1 | 10 | None |  |  |
| MI0006375 | hsa-mir-548f-2 | 2 | rs16848535(A/C) |  |  |
| MI0006376 | hsa-mir-548f-3 | 5 | rs10051407(A/C) |  |  |
| MI0006377 | hsa-mir-548f-4 | 7 | rs826811(C/T) | rs12531159(A/G) | rs826814(C/T) |
|  |  |  | rs826813(C/T) | rs826812(G/T) | rs9648846(A/G) |
|  |  |  | rs6945709(G/T) | rs4142824(C/T) |  |
| MI0006378 | hsa-mir-548f-5 | X | None |  |  |
| MI0006379 | hsa-mir-1244-1 | 12 | rs2070936(C/T) |  |  |
| MI0006380 | hsa-mir-1245a | 2 | rs1878201(A/G) | rs2351420(A/C) |  |
| MI0006381 | hsa-mir-1246 | 2 | rs7585769(A/G) |  |  |
| MI0006382 | hsa-mir-1247 | 14 | None |  |  |
| MI0006383 | hsa-mir-1248 | 3 | rs1426810(C/T) | rs3816112(C/T) | rs266718(C/T) |
| MI0006384 | hsa-mir-1249 | 22 | rs2235160(A/G) | rs5766533(C/T) | rs732373(C/T) |
| MI0006385 | hsa-mir-1250 | 17 | None |  |  |
| MI0006386 | hsa-mir-1251 | 12 | rs6538806(C/T) | rs1582336(C/T) | rs7138358(C/T) |
|  |  |  | rs1579201(C/T) | rs7305831(C/T) | rs17027055(C/T) |
| MI0006387 | hsa-mir-1253 | 17 | rs11871896(A/T) |  |  |
| MI0006388 | hsa-mir-1254-1 | 10 | rs1566222(A/T) | rs10823256(A/C) |  |
| MI0006389 | hsa-mir-1255a | 4 | rs2583394(A/G) | rs1348161(A/G) |  |
| MI0006390 | hsa-mir-1256 | 1 | None |  |  |
| MI0006391 | hsa-mir-1257 | 20 | rs6142899(A/G) |  |  |
| MI0006392 | hsa-mir-1258 | 2 | None |  |  |
| MI0006394 | hsa-mir-1260a | 14 | None |  |  |
| MI0006395 | hsa-mir-548g | 4 | None |  |  |
| MI0006396 | hsa-mir-1261 | 11 | None |  |  |
| MI0006397 | hsa-mir-1262 | 1 | None |  |  |
| MI0006398 | hsa-mir-1263 | 3 | rs12495977(C/T) | rs10936412(C/T) | rs12488423(A/T) |
| MI0006399 | hsa-mir-548n | 7 | rs2557768(C/T) | rs1637696(A/C) | rs1649215(C/T) |
| MI0006400 | hsa-mir-548m | X | None |  |  |
| MI0006401 | hsa-mir-1265 | 10 | rs4850518(A/G) | rs10796190(A/G) | rs11259094(A/G) |
|  |  |  | rs10906675(A/G) | rs10796191(C/T) | rs7896998(C/T) |
|  |  |  | rs10906676(A/G) | rs10906674(C/T) | rs7098908(A/G) |
| MI0006402 | hsa-mir-548o | 7 | None |  |  |
| MI0006403 | hsa-mir-1266 | 15 | rs17650694(C/G) |  |  |
| MI0006404 | hsa-mir-1267 | 13 | None |  |  |
| MI0006405 | hsa-mir-1268a | 15 | None |  |  |
| MI0006406 | hsa-mir-1269a | 4 | rs1397753(C/G) | rs1397755(C/T) | rs977773(C/T) |
|  |  |  | rs17082199(A/G) | rs17082198(A/G) | rs7437072(G/T) |
| MI0006407 | hsa-mir-1270-1 | 19 | None |  |  |
| MI0006408 | hsa-mir-1272 | 15 | None |  |  |
| MI0006409 | hsa-mir-1273a | 8 | None |  |  |
| MI0006411 | hsa-mir-548h-1 | 14 | None |  |  |
| MI0006412 | hsa-mir-548h-2 | 16 | None |  |  |
| MI0006413 | hsa-mir-548h-3 | 17 | rs16948066(C/T) | rs8082242(C/T) | rs8081884(A/G) |
|  |  |  | rs7222005(C/T) | rs7220804(C/T) |  |
| MI0006414 | hsa-mir-548h-4 | 8 | rs7818664(C/G) | rs17362506(C/T) | rs7818211(A/G) |
|  |  |  | rs17056715(A/G) | rs10503808(C/G) | rs7823902(C/T) |
| MI0006415 | hsa-mir-1275 | 6 | None |  |  |
| MI0006416 | hsa-mir-1276 | 15 | None |  |  |
| MI0006417 | hsa-mir-302e | 11 | None |  |  |
| MI0006418 | hsa-mir-302f | 18 | None |  |  |
| MI0006419 | hsa-mir-1277 | X | rs5956962(A/C) |  |  |
| MI0006420 | hsa-mir-548p | 5 | None |  |  |
| MI0006421 | hsa-mir-548i-1 | 3 | None |  |  |
| MI0006422 | hsa-mir-548i-2 | 4 | rs10013288(A/T) | rs9994111(A/G) |  |
| MI0006423 | hsa-mir-548i-3 | 8 | None |  |  |
| MI0006424 | hsa-mir-548i-4 | X | rs6622977(A/G) | rs5968275(C/G) |  |
| MI0006425 | hsa-mir-1278 | 1 | rs10921318(A/G) | rs12126529(A/G) |  |
| MI0006426 | hsa-mir-1279 | 12 | rs1463335(A/T) |  |  |
| MI0006428 | hsa-mir-1281 | 22 | rs5995992(C/T) | rs4822002(A/G) |  |
| MI0006429 | hsa-mir-1282 | 15 | None |  |  |
| MI0006430 | hsa-mir-1283-2 | 19 | rs683060(G/T) |  |  |
| MI0006431 | hsa-mir-1284 | 3 | None |  |  |
| MI0006432 | hsa-mir-1288 | 17 | rs2109262(A/C) | rs3785626(A/C) |  |
| MI0006433 | hsa-mir-1292 | 20 | rs1883977(A/G) | rs4365539(A/T) |  |
|  |  |  | rs4815466(A/G) | rs4813587(C/T) |  |
| MI0006434 | hsa-mir-1252 | 12 | rs11114112(A/G) | rs17294719(C/T) | rs17041667(A/G) |
| MI0006435 | hsa-mir-1255b-1 | 4 | None |  |  |
| MI0006436 | hsa-mir-1255b-2 | 1 | None |  |  |
| MI0006442 | hsa-mir-664a | 1 | None |  |  |
| MI0006443 | hsa-mir-1306 | 22 | rs17817767(A/G) | rs1558496(C/T) |  |
|  |  |  | rs3827292(C/G) | rs2302497(C/T) |  |
| MI0006444 | hsa-mir-1307 | 10 | rs2292807(A/G) |  |  |
| MI0006648 | hsa-mir-513b | X | None |  |  |
| MI0006649 | hsa-mir-513c | X | rs7881366(A/C) |  |  |
| MI0006652 | hsa-mir-1321 | X | rs5967636(C/T) |  |  |
| MI0006653 | hsa-mir-1322 | 8 | rs7829089(A/G) | rs11776767(C/G) | rs7840785(C/T) |
|  |  |  | rs7846449(C/T) | rs7833128(C/T) | rs7824384(A/C) |
|  |  |  | rs2271357(C/T) | rs10109416(C/G) |  |
| MI0006656 | hsa-mir-1197 | 14 | rs7141987(C/T) | rs7141987(C/T) | rs12435862(A/G) |
| MI0006657 | hsa-mir-1324 | 3 | None |  |  |
| MI0007074 | hsa-mir-1469 | 15 | None |  |  |
| MI0007075 | hsa-mir-1470 | 19 | None |  |  |
| MI0007076 | hsa-mir-1471 | 2 | rs2679181(A/G) | rs2679180(C/T) |  |
| MI0007258 | hsa-mir-1537 | 1 | rs7537736(A/T) | rs3768072(C/T) |  |
| MI0007259 | hsa-mir-1538 | 16 | None |  |  |
| MI0007260 | hsa-mir-1539 | 18 | None |  |  |
| MI0007261 | hsa-mir-103b-1 | 5 | None |  |  |
| MI0007262 | hsa-mir-103b-2 | 20 | None |  |  |
| MI0008190 | hsa-mir-320d-1 | 13 | None |  |  |
| MI0008191 | hsa-mir-320c-2 | 18 | rs7239622(C/T) | rs2588567(C/G) |  |
| MI0008192 | hsa-mir-320d-2 | X | rs7886651(A/C) | rs1948488(A/C) |  |
| MI0008193 | hsa-mir-1825 | 20 | None |  |  |
| MI0008195 | hsa-mir-1827 | 12 | None |  |  |
| MI0008329 | hsa-mir-1908 | 11 | None |  |  |
| MI0008330 | hsa-mir-1909 | 19 | rs2304612(C/T) |  |  |
| MI0008331 | hsa-mir-1910 | 16 | rs372395(C/T) |  |  |
| MI0008332 | hsa-mir-1911 | X | rs4911871(A/G) |  |  |
| MI0008333 | hsa-mir-1912 | X | rs12836771(A/G) | rs7887868(C/T) |  |
| MI0008334 | hsa-mir-1913 | 6 | rs3799670(A/G) | rs395475(C/T) | rs3757193(A/G) |
| MI0008335 | hsa-mir-1914 | 20 | rs817370(C/T) |  |  |
| MI0008336 | hsa-mir-1915 | 10 | None |  |  |
| MI0009982 | hsa-mir-1972-1 | 16 | None |  |  |
| MI0009983 | hsa-mir-1973 | 4 | None |  |  |
| MI0009986 | hsa-mir-1976 | 1 | None |  |  |
|  |  |  |  |  |  |
| * MAFs of all the SNPs listed in the table are more than 0.05 in CHB | | | |  |  |
