# Supplementary material for: Genetic variants in microRNAs predict non-small cell lung cancer prognosis in Chinese female population in a prospective cohort study
Source: Oncotarget. 2016 Nov 4;7(50):83101–14. doi: 10.18632/oncotarget.13072 (PMC5347756; doi:10.18632/oncotarget.13072)
Supplement: Supplementary file 3 [file oncotarget-07-83101-s003.docx]

Supplemetary Table 6. The potential targets of miR-149 in Starbase

| Gene | targetScan | picTar | RNA22 | PITA | miRanda | Sum | Position |
| --- | --- | --- | --- | --- | --- | --- | --- |
| TOP1 | 1 | 1 | 1 | 1 | 1 | 5 | chr20:39752102-39752108[+] |
| BCL2L2 | 1 | 1 |  | 1 | 1 | 4 | chr14:23779524-23779531[+] |
| SF1 | 1 | 1 |  | 1 | 1 | 4 | chr11:64533206-64533213[-] |
| CNIH4 | 1 |  | 1 | 1 | 1 | 4 | chr1:224563576-224563583[+] |
| RNF2 | 1 | 1 |  | 1 | 1 | 4 | chr1:185069551-185069558[+] |
| IGF2BP1 | 1 | 1 |  | 1 | 1 | 4 | chr17:47128658-47128664[+] |
| LMBR1L | 1 | 1 |  | 1 | 1 | 4 | chr12:49491295-49491302[-] |
| ATP2A2 | 1 | 1 |  | 1 | 1 | 4 | chr12:110784583-110784589[+] |
| CDK17 | 1 | 1 |  |  | 1 | 3 | chr12:96673789-96673795[-] |
| CHMP7 | 1 |  |  | 1 | 1 | 3 | chr8:23119073-23119079[+] |
| PHLPP2 | 1 | 1 |  |  | 1 | 3 | chr16:71678918-71678925[-] |
| DYNLL2 |  |  | 1 | 1 | 1 | 3 | chr17:56166701-56166722[+] |
| KHSRP |  | 1 |  | 1 | 1 | 3 | chr19:6413880-6413885[-] |
| LONRF1 |  | 1 |  | 1 | 1 | 3 | chr8:12580549-12580554[-] |
| PLEKHJ1 |  |  | 1 |  | 1 | 2 | chr19:2233299-2233320[-] |
| SEC23A |  | 1 |  |  | 1 | 2 | chr14:39501371-39501376[-] |
| MAZ |  |  |  | 1 | 1 | 2 | chr16:29822195-29822201[+] |
| SHMT2 | 1 |  |  |  | 1 | 2 | chr12:57628441-57628447[+] |
| GOLPH3 |  |  |  | 1 | 1 | 2 | chr5:32125697-32125703[-] |
| RNF138 | 1 |  |  |  | 1 | 2 | chr18:29709561-29709567[+] |
| CLK2 |  |  | 1 |  | 1 | 2 | chr1:155232918-155232939[-] |
| NDUFB2 |  |  |  | 1 | 1 | 2 | chr7:140406363-140406369[+] |
| MAP7D1 | 1 |  |  |  | 1 | 2 | chr1:36646030-36646037[+] |
| C20orf4 | 1 |  |  |  | 1 | 2 | chr20:34843792-34843799[+] |
| SRF | 1 |  |  | 1 |  | 2 | chr6:43148205-43148212[+] |
| SERF2 |  | 1 |  | 1 |  | 2 | chr15:44086021-44086026[+] |
| EIF5 | 1 | 1 |  |  |  | 2 | chr14:103807446-103807452[+] |
| NDUFC1 |  |  |  | 1 | 1 | 2 | chr4:140211146-140211170[-] |
| CEP120 |  |  |  |  | 1 | 1 | chr5:122681860-122681882[-] |
| CUL4A |  |  |  |  | 1 | 1 | chr13:113918368-113918393[+] |
| BACH1 |  |  |  |  | 1 | 1 | chr21:30717392-30717414[+] |
| ALDH9A1 |  |  |  |  | 1 | 1 | chr1:165632031-165632056[-] |
| TNFRSF12A | |  |  |  | 1 | 1 | chr16:3071869-3071891[+] |
| NET1 |  |  |  |  | 1 | 1 | chr10:5499741-5499763[+] |
| FAT1 |  |  |  |  | 1 | 1 | chr4:187508981-187509006[-] |
| PDCD6 |  |  |  |  | 1 | 1 | chr5:314689-314712[+] |
| PANK3 |  |  |  |  | 1 | 1 | chr5:167983615-167983637[-] |
| ANKRD11 |  |  |  |  | 1 | 1 | chr16:89334213-89334237[-] |
| SLC35A4 |  |  |  |  | 1 | 1 | chr5:139947850-139947873[+] |
| C9orf40 |  |  |  |  | 1 | 1 | chr9:77561567-77561589[-] |
| APP |  |  |  |  | 1 | 1 | chr21:27253656-27253678[-] |
| RPLP0 |  |  |  |  | 1 | 1 | chr12:120634548-120634570[-] |
| FOXC1 |  |  |  |  | 1 | 1 | chr6:1613494-1613516[+] |
| PANX1 |  |  |  |  | 1 | 1 | chr11:93914961-93914984[+] |
| MAPK6 |  |  |  |  | 1 | 1 | chr15:52357741-52357763[+] |
| PPIA |  |  |  |  | 1 | 1 | chr7:44841659-44841681[+] |
| EGLN3 |  |  |  |  | 1 | 1 | chr14:34394789-34394814[-] |
| CENPB |  |  |  |  | 1 | 1 | chr20:3765240-3765262[-] |
| TSC22D2 |  |  |  |  | 1 | 1 | chr3:150176783-150176805[+] |
| CSTB |  |  |  |  | 1 | 1 | chr21:45194052-45194074[-] |
| PDIA6 |  |  |  |  | 1 | 1 | chr2:10924337-10924359[-] |
| TUBB |  |  |  |  | 1 | 1 | chr6:30693156-30693178[+] |
| C5orf15 |  |  |  |  | 1 | 1 | chr5:133291401-133291426[-] |
| NF1 |  |  |  |  | 1 | 1 | chr17:29687630-29687652[+] |
| CDKN1B |  |  |  |  | 1 | 1 | chr12:12875201-12875223[+] |
| TAF15 |  |  |  |  | 1 | 1 | chr17:34147370-34147392[+] |
| POFUT1 |  |  |  |  | 1 | 1 | chr20:30824740-30824762[+] |
| ARPC1A |  |  |  |  | 1 | 1 | chr7:98935841-98935863[+] |
| TCERG1 |  |  |  |  | 1 | 1 | chr5:145890237-145890258[+] |
| LDHA |  |  |  |  | 1 | 1 | chr11:18429105-18429127[+] |
| ZNF274 |  |  |  |  | 1 | 1 | chr19:58724795-58724817[+] |
| ZCCHC3 |  |  |  |  | 1 | 1 | chr20:279459-279479[+] |
| C14orf43 |  |  |  |  | 1 | 1 | chr14:74182187-74182210[-] |
| CASP3 |  |  |  |  | 1 | 1 | chr4:185549607-185549629[-] |
| KIAA0494 |  |  |  |  | 1 | 1 | chr1:47141950-47141972[-] |
| MKNK2 |  |  |  |  | 1 | 1 | chr19:2037550-2037573[-] |
| FAM168B |  |  |  |  | 1 | 1 | chr2:131805536-131805558[-] |
| MTF2 |  |  |  |  | 1 | 1 | chr1:93603094-93603116[+] |
| PGK1 |  |  |  |  | 1 | 1 | chrX:77369345-77369366[+] |
| DNAJB9 |  |  |  |  | 1 | 1 | chr7:108214625-108214647[+] |
| CHD1 |  |  |  |  | 1 | 1 | chr5:98191532-98191554[-] |
| MLLT3 |  |  |  |  | 1 | 1 | chr9:20346266-20346288[-] |
| DCUN1D5 |  |  |  |  | 1 | 1 | chr11:102932907-102932930[-] |
| GPR63 |  |  |  |  | 1 | 1 | chr6:97245921-97245944[-] |
| EIF2C4 |  |  |  |  | 1 | 1 | chr1:36319365-36319388[+] |
| ATL2 |  |  |  |  | 1 | 1 | chr2:38522692-38522717[-] |
| ARID4B |  |  |  |  | 1 | 1 | chr1:235331054-235331076[-] |
| NDUFA7 |  |  |  |  | 1 | 1 | chr19:8376295-8376318[-] |
| LASS2 |  |  |  |  | 1 | 1 | chr1:150937845-150937867[-] |
| SOLH |  |  |  |  | 1 | 1 | chr16:603588-603611[+] |
| HNRNPUL1 |  |  |  |  | 1 | 1 | chr19:41813102-41813121[+] |
| SDCBP |  |  |  | 1 |  | 1 | chr8:59494384-59494391[+] |
| ID3 |  |  |  | 1 |  | 1 | chr1:23884729-23884735[-] |
| CDKN1A |  |  | 1 |  |  | 1 | chr6:36654736-36654757[+] |
| STXBP5 |  |  | 1 |  |  | 1 | chr6:147706273-147706294[+] |
| PRPSAP1 |  |  | 1 |  |  | 1 | chr17:74307511-74307532[-] |
| TMEM127 |  |  | 1 |  |  | 1 | chr2:96919282-96919303[-] |
| ZBTB39 |  |  | 1 |  |  | 1 | chr12:57395961-57395982[-] |
| CEBPG |  |  | 1 |  |  | 1 | chr19:33872151-33872172[+] |
| CORO1C |  |  | 1 |  |  | 1 | chr12:109039505-109039526[-] |
| WSB1 |  |  | 1 |  |  | 1 | chr17:25639768-25639789[+] |
| PSME3 |  |  | 1 |  |  | 1 | chr17:40994981-40995002[+] |
| EZH1 |  |  | 1 |  |  | 1 | chr17:40854352-40854373[-] |
| EIF3G |  |  | 1 |  |  | 1 | chr19:10225915-10225936[+] |
| PLXNA1 |  |  | 1 |  |  | 1 | chr3:126755938-126755959[+] |
| TXNIP |  |  | 1 |  |  | 1 | chr1:145442457-145442478[+] |
| CLIP4 |  |  | 1 |  |  | 1 | chr2:29405217-29405238[+] |
| EPDR1 |  |  | 1 |  |  | 1 | chr7:37990794-37990815[+] |
| TOMM40 |  |  | 1 |  |  | 1 | chr19:45406702-45406723[+] |
| SLC9A1 |  |  | 1 |  |  | 1 | chr1:27425512-27425533[-] |
| TXLNA |  |  | 1 |  |  | 1 | chr1:32663812-32663833[+] |
| FBXW11 |  |  | 1 |  |  | 1 | chr5:171290315-171290336[-] |
| STARD7 |  |  | 1 |  |  | 1 | chr2:96850888-96850909[-] |
| ZNF529 |  |  | 1 |  |  | 1 | chr19:37037356-37037377[-] |
| USP6NL |  |  | 1 |  |  | 1 | chr10:11502827-11502848[-] |
| NFIB |  | 1 |  |  |  | 1 | chr9:14087104-14087109[-] |
| KIAA0317 |  | 1 |  |  |  | 1 | chr14:75130320-75130325[-] |
| BCL2L11 |  | 1 |  |  |  | 1 | chr2:111924489-111924494[+] |
| CTCF |  | 1 |  |  |  | 1 |  |
| SP1 |  | 1 |  |  |  | 1 | chr12:53808958-53808963[+] |
| KIF2A |  | 1 |  |  |  | 1 | chr5:61681421-61681426[+] |
| FZD5 |  | 1 |  |  |  | 1 | chr2:208627840-208627845[-] |
| BTG2 |  | 1 |  |  |  | 1 | chr1:203278178-203278183[+] |
| PPM1F |  | 1 |  |  |  | 1 | chr22:22274600-22274605[-] |
| BRPF3 |  | 1 |  |  |  | 1 | chr6:36199008-36199014[+] |
| MLL2 |  | 1 |  |  |  | 1 | chr12:49415252-49415257[-] |
| HMGA2 |  | 1 |  |  |  | 1 | chr12:66358295-66358300[+] |
| MAP3K2 |  | 1 |  |  |  | 1 | chr2:128065148-128065153[-] |
| EIF4A1 |  | 1 |  |  |  | 1 | chr17:7481883-7481888[+] |
| REEP3 | 1 |  |  |  |  | 1 | chr10:65383424-65383431[+] |
| GATAD2B | 1 |  |  |  |  | 1 | chr1:153780188-153780194[-] |
| M6PR | 1 |  |  |  |  | 1 | chr12:9094354-9094360[-] |
