# Supplementary material for: Genetic variants in microRNAs predict non-small cell lung cancer prognosis in Chinese female population in a prospective cohort study
Source: Oncotarget. 2016 Nov 4;7(50):83101–14. doi: 10.18632/oncotarget.13072 (PMC5347756; doi:10.18632/oncotarget.13072)
Supplement: Supplementary file 4 [file oncotarget-07-83101-s004.docx]

Supplementary Table 7. The fold change no less than 1.3 for mRNAs between lung cancer and normal tissues.

| geneName | AD FC | SCC FC |
| --- | --- | --- |
| HMGA2 | 7.766688 | 54.70301 |
| IGF2BP1 | 6.74808 | 21.28862 |
| EGLN3 | 4.796695 | 7.514872 |
| SHMT2 | 2.603698 | 3.895647 |
| DCUN1D5 | 1.7 | 2.9 |
| MAPK6 | 1.397751 | 2.797236 |
| LDHA | 2.441293 | 2.566169 |
| TOMM40 | 1.658472 | 2.504666 |
| TUBB | 1.599591 | 2.39404 |
| NET1 | 2.238107 | 2.376291 |
| PGK1 | 1.618948 | 2.277759 |
| PDCD6 | 1.978049 | 2.230045 |
| MAZ | 1.561326 | 2.165431 |
| PDIA6 | 2.022395 | 2.139296 |
| ATP2A2 | 1.612919 | 1.936226 |
| RPLP0 | 1.57 | 1.93 |
| CEBPG |  | 1.912251 |
| C9orf40 | 1.557346 | 1.90795 |
| FAT1 | 1.7 | 1.9 |
| CASP3 | 1.645503 | 1.893049 |
| ARPC1A | 1.429038 | 1.879761 |
| POFUT1 |  | 1.741156 |
| CLK2 | 1.907538 | 1.726141 |
| ATL2 | 1.484997 | 1.722032 |
| CSTB |  | 1.681601 |
| RNF2 |  | 1.676377 |
| BCL2L11 | 1.387396 | 1.674285 |
| PLXNA1 |  | 1.63253 |
| PSME3 | 1.373273 | 1.628644 |
| ZBTB39 |  | 1.585549 |
| PANK3 |  | 1.569778 |
| TCERG1 | 1.508221 | 1.56178 |
| PPIA | 1.412927 | 1.557163 |
| SOLH | 1.571403 | 1.480274 |
| KIF2A | 1.581286 | 1.475026 |
| PANX1 | 1.446235 | 1.451479 |
| GPR63 |  | 1.435766 |
| EIF4A1 |  | 1.392154 |
| BRPF3 | 1.566097 |  |
| TNFRSF12A | 1.378709 |  |
| LMBR1L | 1.36 |  |
| REEP3 | 1.359724 |  |
| TOP1 | 1.359096 |  |
| SEC23A | 1.322512 |  |
| NF1 | 1.317933 |  |
| GOLPH3 | 1.308599 |  |
