# Supplementary material for: Genetic variants in microRNAs predict non-small cell lung cancer prognosis in Chinese female population in a prospective cohort study
Source: Oncotarget. 2016 Nov 4;7(50):83101–14. doi: 10.18632/oncotarget.13072 (PMC5347756; doi:10.18632/oncotarget.13072)
Supplement: Supplementary file 5 [file oncotarget-07-83101-s005.docx]

Supplementary Table 8. GO and KEGG analyses for TOP1

| #pathway ID | pathway description | observed gene count | FDR |
| --- | --- | --- | --- |
| Biological Process (GO) | | | |
| GO.0006265 | DNA topological change | 3 | 0.000134 |
| GO.0044774 | mitotic DNA integrity checkpoint | 4 | 0.00134 |
| GO.0006259 | DNA metabolic process | 6 | 0.00183 |
| GO.0045786 | negative regulation of cell cycle | 5 | 0.00362 |
| GO.0071103 | DNA conformation change | 4 | 0.00577 |
| GO.0040016 | embryonic cleavage | 2 | 0.0076 |
| GO.0034349 | glial cell apoptotic process | 2 | 0.00921 |
| GO.0000712 | resolution of meiotic recombination intermediates | 2 | 0.0109 |
| GO.0006268 | DNA unwinding involved in DNA replication | 2 | 0.0109 |
| GO.0006974 | cellular response to DNA damage stimulus | 5 | 0.0109 |
| GO.0048511 | rhythmic process | 4 | 0.0109 |
| GO.0051307 | meiotic chromosome separation | 2 | 0.0149 |
| GO.0007623 | circadian rhythm | 3 | 0.0373 |
| Molecular Function(GO) | | | |
| GO.0003918 | DNA topoisomerase type II (ATP-hydrolyzing) activity | 3 | 9.51E-07 |
| GO.0003916 | DNA topoisomerase activity | 2 | 0.0027 |
| GO.0008022 | protein C-terminus binding | 3 | 0.0313 |
| GO.0008301 | DNA binding, bending | 2 | 0.0341 |
| GO.0003682 | chromatin binding | 4 | 0.0435 |
| Cellular Component(GO) | | | |
| GO.0009330 | DNA topoisomerase complex (ATP-hydrolyzing) | 2 | 0.00021 |
| GO.0036464 | cytoplasmic ribonucleoprotein granule | 4 | 0.00021 |
| GO.0000932 | cytoplasmic mRNA processing body | 3 | 0.00212 |
| GO.0005654 | nucleoplasm | 8 | 0.00263 |
| GO.0030529 | ribonucleoprotein complex | 5 | 0.00263 |
| GO.0032991 | macromolecular complex | 9 | 0.00534 |
| GO.0031981 | nuclear lumen | 8 | 0.00695 |
| GO.0005737 | cytoplasm | 11 | 0.0198 |
| GO.0005657 | replication fork | 2 | 0.0328 |
| KEGG pathway | | | |
| 5168 | Herpes simplex infection | 3 | 0.0272 |
| 5014 | Amyotrophic lateral sclerosis (ALS) | 2 | 0.0395 |
| 5210 | Colorectal cancer | 2 | 0.0395 |
| 4115 | p53 signaling pathway | 2 | 0.0397 |
